# Human long noncoding RNA *VILMIR* is induced by major respiratory viral infections and modulates the host interferon response

**DOI:** 10.1128/jvi.00141-25

**Published:** 2025-03-25

**Authors:** Kristen John, Ian Huntress, Ethan Smith, Hsuan Chou, Tammy S. Tollison, Sergio Covarrubias, Elisa Crisci, Susan Carpenter, Xinxia Peng

**Affiliations:** 1 Department of Molecular Biomedical Sciences, North Carolina State University College of Veterinary Medicine 70727 , Raleigh, North Carolina, USA; 2 Genetics & Genomics Graduate Program, North Carolina State University 6798 , Raleigh, North Carolina, USA; 3 Bioinformatics Graduate Program, North Carolina State University 6798 , Raleigh, North Carolina, USA; 4 Department of Molecular, Cell and Developmental Biology, University of California Santa Cruz 214546 , Santa Cruz, California, USA; 5 Department of Population Health and Pathobiology, North Carolina State University College of Veterinary Medicine 70727 , Raleigh, North Carolina, USA; 6 Bioinformatics Research Center, North Carolina State University 169132 , Raleigh, North Carolina, USA; Loyola University Chicago - Health Sciences Campus, Maywood, Illinois, USA

**Keywords:** interferon, lncRNA, influenza, SARS-CoV-2, RSV, ISG, RNA-seq

## Abstract

Long noncoding RNAs (lncRNAs) are a newer class of noncoding transcripts identified as key regulators of biological processes. Here, we aimed to identify novel lncRNA targets that play critical roles in major human respiratory viral infections by systematically mining large-scale transcriptomic data sets. Using bulk RNA-sequencing (RNA-seq) analysis, we identified a previously uncharacterized lncRNA, named virus-inducible lncRNA modulator of interferon response (*VILMIR*), that was consistently upregulated after *in vitro* influenza infection across multiple human epithelial cell lines and influenza A virus subtypes. *VILMIR* was also upregulated after severe acute respiratory syndrome coronavirus 2 (SARS-CoV-2) and respiratory syncytial virus (RSV) infections *in vitro*. We experimentally confirmed the response of *VILMIR* to influenza infection and interferon-beta (IFN-β) treatment in the A549 human epithelial cell line and found the expression of *VILMIR* was robustly induced by IFN-β treatment in a dose- and time-specific manner. Single-cell RNA-seq analysis of bronchoalveolar lavage fluid samples from coronavirus disease 2019 (COVID-19) patients uncovered that *VILMIR* was upregulated across various cell types, including at least five immune cells. The upregulation of *VILMIR* in immune cells was further confirmed in the human T cell and monocyte cell lines, SUP-T1 and THP-1, after IFN-β treatment. Finally, we found that knockdown of *VILMIR* expression reduced the magnitude of host transcriptional responses to both IFN-β treatment and influenza A virus infection in A549 cells. Together, our results show that *VILMIR* is a novel interferon-stimulated gene (ISG) that regulates the host interferon response and may be a potential therapeutic target for human respiratory viral infections upon further mechanistic investigation.

**IMPORTANCE:**

Identifying host factors that regulate the immune response to human respiratory viral infection is critical to developing new therapeutics. Human long noncoding RNAs (lncRNAs) have been found to play key regulatory roles during biological processes; however, the majority of lncRNA functions within the host antiviral response remain unknown. In this study, we identified that a previously uncharacterized lncRNA, virus-inducible lncRNA modulator of interferon response (*VILMIR*), is upregulated after major respiratory viral infections including influenza, severe acute respiratory syndrome coronavirus 2, and respiratory syncytial virus. We demonstrated that *VILMIR* is an interferon-stimulated gene that is upregulated after interferon-beta (IFN-β) in several human cell types. We also found that knockdown of *VILMIR* reduced the magnitude of host transcriptional responses to IFN-β treatment and influenza A infection in human epithelial cells. Our results reveal that *VILMIR* regulates the host interferon response and may present a new therapeutic target during human respiratory viral infections.

## INTRODUCTION

With the first draft of the human genome, researchers discovered that protein-coding genes only account for less than 2% of the genome (1). At first glance, the remaining 98% of intergenic sequence was considered “junk DNA.” However, advances in next-generation sequencing technology revealed that about 80% of the genome may be transcribed, the majority of which is noncoding RNAs (ncRNAs) (2). While some ncRNAs are well characterized, such as microRNAs, much less is known about the function of long noncoding RNAs (lncRNAs). LncRNAs are transcripts greater than 500 nucleotides in length that have low translational potential (3). They are the largest class of ncRNAs with 20,424 human lncRNA genes annotated in the recent GENCODE V45 release (4); however, identifying their functions has been challenging due to their low expression levels and low evolutionary conservation at the sequence level (5, 6). Despite these challenges, lncRNAs have emerged as key regulators of biological processes, such as transcription, mRNA stabilization, and protein translation (7). More recently, multiple lncRNAs have been identified in host antiviral and immune responses (8–10).

LncRNAs were first suggested to regulate viral infections when they were differentially expressed in mice after both severe acute respiratory syndrome coronavirus (SARS-CoV) and influenza infections (11). While RNA-sequencing (RNA-seq) analysis identified over 5,000 lncRNAs related to these viral infections, their specific roles in infection were not explored (12). Since then, individual lncRNAs have been studied in more detail and found to significantly alter influenza infection in human epithelial cells by either inhibiting or promoting viral infection (13). For example, lncRNA lnc-MxA was found to promote influenza A virus (IAV) infection by forming an RNA-DNA triplex at the interferon-beta (IFN-β) promoter and inhibiting the transcription of IFN-β (14). Lnc-ISG20 was found to inhibit IAV infection by competitively binding to microRNA 326 and reducing its inhibition of ISG20 translation (15). While a few IFN-independent lncRNAs have been identified in influenza infection (16–19), many of the identified lncRNAs within influenza infection are regulated by the IFN pathway to either promote the host immune response or control the immune response through negative feedback mechanisms (13).

In this study, we mined large-scale public RNA-seq data and identified a previously uncharacterized human lncRNA that we found was consistently upregulated after IAV infection across multiple human epithelial cell lines and influenza A virus subtypes. We showed that this lncRNA, virus-inducible lncRNA modulator of interferon response (*VILMIR*), was also upregulated after SARS-CoV-2 infection, respiratory syncytial virus (RSV) infection, and after IFN-β treatment. In addition, we analyzed single-cell RNA-seq data from bronchoalveolar lavage fluid (BALF) samples from coronavirus disease 2019 (COVID-19) patients and demonstrated that *VILMIR* was upregulated in multiple cell types including epithelial cells and at least five immune cell types. Finally, we found that knockdown (KD) of *VILMIR* in human epithelial A549 cells broadly dampened the host response to IFN-β treatment and IAV infection. Our results show that lncRNA *VILMIR* is a novel IFN-stimulated gene (ISG) responding to major respiratory viral infections and may play a broad role in antiviral and innate immunity requiring further mechanistic investigation.

## MATERIALS AND METHODS

### RNA-sequencing data analysis

From public influenza infection experiments made available through the Gene Expression Omnibus (20, 21), we collected 121 bulk RNA-seq samples. These samples included four human epithelial cell lines from the respiratory tract and 10 different influenza strains, and they ranged from 1 to 48 hours post influenza infection (summarized in Table 1). Infected and control samples were selected from experiments GSE97949 (22), GSE75699 (23, 24), GSE89008 (25), GSE68673 (26), GSE61517 (27), and GSE104168 (28). Samples were mapped using STAR version 2.5.2b (29) to the human genome assembly 38 (Hg38) with GENCODE V25. Custom STAR parameters were set as follows: twopassMode: Basic and alignIntronMax: 100,000; otherwise, default STAR parameters were used. Raw gene counts were filtered, requiring at least one sample with greater than 50 reads. Remaining gene counts were normalized using the voom quantile normalization method (30) from the R package Limma (31). Each unique infected condition was compared to the time, strain, and cell type-matched controls defined in their public experiment, for a total of 32 contrasts or comparisons. A gene was considered significantly differentially expressed for a comparison if its unadjusted *P*-value was less than 0.05 and the absolute value of the log2 fold change (log2FC) was greater than 0.58, i.e., a minimum change of 1.5-fold.

**TABLE 1:** Summary of the collection of RNA-seq data from influenza infections in human epithelial cells as seen in Fig. 1

Epithelial cell line	IAV subtype	Strain	Abbr.	MOI^*a*^	Time points	Ref.
A549 (adenocarcinomic human alveolar basal epithelial cells)	H1N1*^b^	A/Wisconsin/33	*^b^	3	1	(26)
	H1N1	A/Puerto Rico/8/1934	PR8	0.5	2	(28)
	H3N2	A/New York/238/2005	NY05		2	
	H7N9	A/Anhui/1/2013	A13	1	3	(22)
BEAS-2B (epithelial cells from normal human lung epithelium)	H3N2	A/Brisbane/10/07	B07	1	3	(27)
	H3N2	A/Perth/16/09	P09			
	H3N2	A/Udorn/307/72	U72			
Human tracheobronchial epithelial (HTBE) cells	H1N1	A/California/04/09	CA09	5	5	(25)
	H3N2	A/Wyoming/03/03	WY03		4	
	H5N1	A/Vietnam/1203/04^*c*^	V04		4	
Primary normal human bronchial epithelial (NHBE) cells	H1N1	A/Puerto Rico/8/1934	PR8	1	2	(23, 24)

^*a*^ MOI, multiplicity of infection.

^*b*^ Unclear from reference 26 which one of the three viruses was used: A/Wisconsin/33 (WSN), A/New Caledonia/20/99, or a recombinant virus.

^*c*^ Mutant H5N1 as described in reference 32.

To select lncRNAs that were more relevant to IAV infection, we applied three major selection criteria. (i) Genes were consistently detected across all but one of the human epithelial cell lines (i.e., one count per million reads mapped in all the uninfected control groups or one of the infection groups for at least one time point). (ii) The same gene consistently exhibited large expression changes when the overall host response peaked during infection (upregulation or downregulation of fourfold or more in at least 5 of 10 peak time points). (iii) The same gene exhibited significant expression changes early after infection (6–7 hours after infection) with a fold change of 1.5 or more in at least six of nine early time points (to account for smaller and more variable expression changes at earlier time points).

To investigate if *VILMIR* was differentially expressed under additional conditions, we separately analyzed 78 single-end RNA-seq samples (120 to 140 bp read length) from GSE147507 (33, 34). For this analysis, reads were mapped using STAR version 2.7.10b (29) to the Hg38 with GENCODE version 44. Lowly expressed genes were filtered out using the filterByExpr function in edgeR version 3.40.2 (35) with default parameters. Counts were normalized using the trimmed mean of M values (TMM) normalization method via the calcNormFactors function in edgeR. Differentially expressed genes (DEGs) were identified using the voom method (30) of the Limma R package version 3.54.2 (31). To calculate gene log fold changes in response to infection, we adopted the same sample comparisons previously defined by Blanco-Melo et al. (33).

### Cell culture

The human cancer cell lines, A549 lung epithelial (CCL-185), SUP-T1 T lymphoblast (CRL-1942), and THP-1 monocyte (TIB-202), were purchased from American Type Culture Collection (ATCC, Manassas, VA, USA). Madin-Darby canine kidney (MDCK) cells (London Line, FR-58) were obtained through the International Reagent Resource, Influenza Division, WHO Collaborating Center for Surveillance, Epidemiology and Control of Influenza, Centers for Disease Control and Prevention, Atlanta, GA, USA. Human embryonic kidney (HEK) epithelial 293FT cells were ordered from Invitrogen. Human wild-type (WT) and IFNAR1 knockout (KO) Huh7 tumorigenic liver cells were a gift from Ram Savan, University of Washington (36). A549 cell lines were maintained in F-12K media with 10% fetal bovine serum (FBS). MDCK cells were maintained in Dulbecco’s modified Eagle medium (DMEM) plus 100 U/mL penicillin and 100 µg/mL streptomycin (P/S), 0.2% bovine albumin fraction V, 25 mM HEPES buffer, and 5% FBS. SUP-T1 cells were maintained in RPMI 1640 media with 1% glutamax, P/S, and 10% FBS. THP-1 cells were maintained in the same media as SUP-T1 except for an additional supplement of 0.05 mM 2-mercaptoethanol. HEK 293FT cells were maintained in DMEM plus 1% glutamax and 10% FBS. Huh7 cells were maintained in DMEM plus P/S and 10% FBS. All cell lines were kept at 37°C in a 5% CO_2_ incubator.

### Influenza virus stocks

Influenza A/California/04/2009 (H1N1) virus was ordered from BEI Resources (NR-13658) and propagated in MDCK cells as previously described by the World Health Organization (37). Briefly, MDCK cells at about 95% confluency were washed twice with 1× Dulbecco’s phosphate-buffered saline (DPBS) and once with serum-free media and then infected with viral inoculum at a multiplicity of infection (MOI) of 0.0001 in serum-free media for 1 hour at 37°C, 5% CO_2_. After 1 hour, additional serum-free media containing 0.2% bovine albumin fraction V and 2 µg/mL N-tosyl-L-phenylalanyl chloromethyl ketone (TPCK)-trypsin was added, and the cells were incubated at 37°C, 5% CO_2_ until cytopathic effect reached at least 75%. The cell culture supernatant was harvested, centrifuged at 500 × *g* for 10 min, and stored at −80°C.

Virus titer was determined by tissue culture infectious dose (TCID_50_) assay using an enzyme-linked immunosorbent assay (ELISA) as described previously (38). Briefly, MDCK cells were incubated with 1/2-log dilutions of virus in serum-free media for 18 hours at 37°C, 5% CO_2_. The next day, the cells were washed with DPBS and fixed in cold 80% acetone for 10 min. Viral nucleoprotein (NP) was detected by ELISA as described using a 1/1,000 dilution of the anti-NP antibody (Millipore cat. #MAB8257) and a 1/2,000 dilution of the horseradish peroxidase-labeled goat anti-mouse IgG (SeraCare cat. #52200460). Freshly prepared substrate (10 mg of σ-phenylenediamine dihydrochloride [Sigma-Aldrich, cat. # P8287] per 20 mL of 0.05 M phosphate citrate buffer, pH 5.0, containing 0.03% sodium perborate) was added to each well, and the reaction was stopped with an equal volume of 0.5 M sulfuric acid. Absorbance was measured at 490 nm using a Tecan Spark microplate reader, and the TCID_50_ was calculated using the Reed-Muench method (39).

### Virus infection

A549 cells were seeded overnight at 150,000 or 175,000 cells per well in 12-well plates in 1.5 mL media to reach ~80%–90% confluency the next day. The cell monolayer was washed twice with 1× DPBS and then incubated with virus at MOI 0.1 or mock inoculum in serum-free media for 1 hour at 37°C, 5% CO_2_. After 1 hour, the inoculum was removed, and the cells were washed again with 1× DPBS. Additional serum-free media containing 0.2% bovine albumin fraction V and 0.5 µg/mL TPCK-trypsin was added, and the cells were incubated at 37°C, 5% CO_2_. At the indicated time points post-infection, RNA was extracted for analysis following the TRIzol RNA isolation method (Invitrogen).

### Interferon treatment

A549 cells were seeded overnight at 150,000 cells per well in 12-well plates in 1.5 mL media to reach ~80% confluency the next day. The media was then removed, and the cell monolayer was washed with 1× DPBS prior to interferon treatment. Cells were treated with fresh A549 media with or without human IFN-β recombinant protein (R&D Systems 8499IF010) at the indicated concentrations. Cells were harvested at the indicated time points after treatment following the TRIzol RNA isolation method (Invitrogen). Huh7 cells were seeded between 200,000 and 250,000 cells in 12-well plates in 1.5 mL and incubated in media with or without human IFN-β at 10 ng/mL for 6 hours and then harvested by TRIzol. SUP-T1 and THP-1 cells were seeded at 1 million cells per well in 12-well plates in 1 mL and incubated in media with or without human IFN-β at 10 ng/mL for 6 hours and then harvested by TRIzol.

### RNA isolation and quantitative PCR

Total RNA was isolated from cells following the TRIzol isolation method (Invitrogen) and quantified using Nanodrop spectrophotometry. One microgram of RNA was reverse-transcribed into cDNA using the QuantiTect Reverse Transcription Kit (Qiagen) containing both oligo-dT and random primers. Quantitative PCR (qPCR) was performed on the cDNA using PowerUp SYBR Green Master Mix (Applied Biosystems). Relative expression of the indicated RNAs was determined using the ΔΔCt method with *GAPDH* or *18S* as an endogenous control. Statistical analysis of significance was performed in JMP Pro 16 software (SAS Institute Inc., Cary, NC). The primer sequences used in this study are as follows: *GAPDH* F: GGTATCGTGGAAGGACTCATGAC; *GAPDH* R: ATGCCAGTGAGCTTCCCGTTCAG (16); *18S* F: GAACGTCTGCCCTATCAACTTTC*; 18S* R: GATGTGGTAGCCGTTTCTCAG; *VILMIR* F: GCTCCACCCTGAAAGTC; *VILMIR* R: CTACACAGTGCTGAGGAAA; *IFN-β* F: GCTCTCCTGTTGTGCTTCTCCAC; *IFN-β* R: CAATAGTCTCATTCCAGCCAGTGC (40); *STAT1* F: ATGCTTGCTTGGATCAGCTG; *STAT1* R: TAGGGTCATGTTCGTAGGTG; *GPB1* F: cgctcttaaacttcaggaacag; *GBP1* R: cgtcgtctcattttcgtctgg; *MALAT1* F: TCCCCACAAGCAACTTCTCT; *MALAT1* R: CCTCGACACCATCGTTACCT; *DANCR* F: CGGAGGTGGATTCTGTTAGG; *DANCR* R: TCGGTGTAGCAAGTCTGGTG; *RHOT1* F: GCTCTGGAGGATGTCAAGAATG; *RHOT1* R: CGTGTCTCCCTCTCTGGATAA; *LRRC37B* F: GGACCTGGAGCTTAGCATAAC; *LRRC37B* R: GTCCAATCTCTGTAGTGGGTTC.

### 5´ and 3´ rapid amplification of cDNA ends (RACE)

5´ RACE of lncRNA *VILMIR* was performed using the SMARTer RACE 5´/3´ Kit (Takara, USA) according to the manufacturer’s instructions. To ensure a high level of expression of *VILMIR*, RNA from IFN-β-treated A549 cells was used for first-strand cDNA synthesis. 5´ RACE PCR was then performed using the 5′-RACE-ready cDNA and a gene-specific primer (GSP) with the sequence GCTCACCACCTGTAATCCCAGTAT. The 5´ RACE product was cloned into the pRACE vector provided by the RACE kit and sequenced with Sanger sequencing.

As obtaining a 3´ RACE product using the SMARTer RACE kit was not successful, a different protocol was used for this purpose. cDNA for 3´ RACE was generated using RNA from IFN-β-treated A549 cells using the Template Switch RT Enzyme Mix (NEB, #M0466) and anchored primers. PCR was performed in two rounds using a nested primer design to enrich for *VILMIR* with forward GSPs (first round: ACGGTTTGGCTGATGGAAGATG, second round: CTCTGTGCTTCTAAACTCACTA) and a reverse primer to the 3´ anchor sequence introduced in RT. PCR was performed using Titanium Taq (Takara, #639208) with an extension time of 90 s for 30 (first round) or 20 (second round) cycles of amplification. PCR products were purified by PAGE using an overnight elution in 0.1× Tris-acetate-EDTA (TAE) on an orbital shaker. Purified round 2 PCR products were cloned using the NEB PCR Cloning Kit (NEB, #E1202) and sequenced with Sanger sequencing.

### Isolation of cytoplasmic and nuclear RNAs

Cytoplasmic, nuclear, and total RNA fractions were prepared from A549 cells according to the RNA Subcellular Isolation Kit (Active Motif, Cat. #25501). cDNA was prepared as described above, and qPCR was performed to analyze *VILMIR* expression in both cellular fractions. Total RNA was used for normalization to calculate the percentage of total RNA by the equation % of Input = 100 × [2^ (Ct total RNA – Ct RNA fraction)]. *MALAT1* and *DANCR* were used as nuclear and cytoplasmic lncRNA controls, respectively.

### Protein extraction and Western blotting

To collect protein lysate for Western blotting, confluent T75 flasks of A549 knockdown cell lines were washed with DPBS and treated with fresh A549 media with or without 10 ng/mL human IFN-β recombinant protein for 6 hours. Approximately 500,000 cells were removed for TRIzol RNA isolation. Nuclear and cytoplasmic protein lysate of the remaining cells was extracted using NE-PER Nuclear and Cytoplasmic Extraction Reagents (Thermo Scientific) according to the manufacturer’s instructions. Relative protein quantification was determined using absorbance at 280 nm by comparison with a bovine serum albumin standard curve.

Protein lysate was loaded into 10% SDS-PAGE gels at 50 µg per sample and transferred to polyvinylidene difluoride (PVDF) membranes (Invitrogen). Membranes were blocked overnight at 4°C in 1× Tris-buffered saline (TBS) with 1% (wt/vol) casein and then incubated in primary antibody diluted in blocking buffer for 1 hour at room temperature. The following primary antibodies were used: anti-STAT1 1:2,000 (Proteintech, 10144-2-AP), anti-Phospho-STAT1 (Tyr701) (58D6) 1:1,000 (Cell Signaling Technology, #9167), anti-PCNA 1:10,000 (Proteintech, 10205-2-AP), and anti-GAPDH 1:10,000 (Proteintech, 10494-1-AP). Membranes were rinsed 2× and washed 2× for 5 min each in TBS buffer with 0.05% Tween 20 (TBS-T) and then incubated in Goat anti-Rabbit IgG (H + L) secondary antibody, horseradish peroxidase (HRP) conjugate (Invitrogen #31460) diluted 1:2,000 in blocking buffer for 30 min at room temperature. Membranes were rinsed 3× and washed 3× for 5 min each in TBS-T buffer and then detected by chemiluminescence using Pierce ECL Substrate (Thermo Scientific). The blots were visualized using a Bio-Rad ChemiDoc MP Imaging System. Densitometry analysis was performed using ImageJ software. Briefly, the background was subtracted, and the signal intensity of each band was measured. The signal of the target protein (STAT1 or p-STAT1) was normalized to the loading control signal from each lane (GAPDH for cytoplasmic and PCNA for nuclear lysate samples). The normalized signal was averaged for three independent replicates.

### Plasmids and cloning

The dCas9-KRAB construct was ordered from Addgene (#89567). The green fluorescent protein (GFP) construct for overexpression was constructed from a pSico lentiviral backbone with a bidirectional minimal EF1A-minimal CMV promoter expressing T2A flanked genes: zeocin-resistant (Zeo) and GFP. The gRNA construct was published previously (41). Guide RNA (gRNA) sequences targeting the transcription start site of *STAT1* and *VILMIR* were designed using the web-based tool, CRISPick by Broad Institute (42, 43). Two highly ranked gRNAs were selected for each gene. The sequences of the gRNAs are as follows: STAT1g1, GGTCGCCTCTGCTCGGTCTG; STAT1g2, GGAGGGGCTCGGCTGCACCG; VILMIRg1, GTCATGCGGAGGACAAGGAA; VILMIRg2, CCTCCGCATGACGCCCGTGC. The sequence of the control gRNA targeting GFP was TGGTGGGCTAGCGGATCTGA. Each pair of gRNA spacer forward/reverse oligos were annealed and ligated into the gRNA construct via the AarI site.

### dCas9-KRAB cell line construction and validation

To produce lentivirus, HEK-293FT cells were co-transfected with each construct described above and lentiviral vectors, psPAX2 (Addgene #12260) and pMD2.G (Addgene #12259) using the Lipofectamine 3000 Reagent (Invitrogen). A549 cells were first lentivirally infected with the dCas9-KRAB construct (described above) and were selected using blasticidin (10 µg/mL) for 1 week to obtain dCas9-KRAB-expressing cells. The same cells were lentivirally infected with the GFP construct and selected using zeocin (200 µg/mL–300 µg/mL) for 1 week. Next, the dCas9-KRAB-GFP-expressing cells were clonally expanded and infected with a GFP-targeting gRNA and selected with puromycin (2 µg/mL–5 µg/mL) for 1 week to establish efficient GFP knockdown using flow cytometry as a readout. *STAT1* and *VILMIR* gRNAs were then inserted into the most efficient dCas9-KRAB-GFP cells and selected to assay the effects of knockdown.

### Single-cell RNA-seq data analysis

We collected nine SARS-CoV-2-infected 5´ single cell RNA-seq samples and three uninfected 5´ single cell RNA-seq samples from Liao et al. (GSE145926) (44). The Cell Ranger Software Suite version 7.0.1 (45) was used to align sequencing reads and generate feature-barcode matrices. Reads were mapped to the Hg38 with GENCODE V45 annotation provided by Ensembl (46). The Seurat R package version 4.3.0 (47) was used to join the cell type annotations from the final barcode-cell type mapping matrix published by Liao et al. (44) to the corresponding cells in the feature-barcode matrices produced by Cell Ranger. Cells that were not assigned an identity by Liao et al. (44) were excluded from downstream analysis. Additionally, cell types that had low relative abundance (<0.15% of annotated infected cells) in the infected samples were removed from the analysis. Cell types that were considered abundant in the infected samples but had low relative abundance in control samples (<0.15% of annotated control cells) were retained but had their control data excluded from the analysis. For cell types with sufficient cell counts in each infected and control sample (>50 cells), a Wilcoxon rank sum test was conducted comparing the percentages of *VILMIR* positive cells in infected vs uninfected samples, separately for each cell type.

### cDNA library construction, RNA-sequencing, and Ingenuity Pathway Analysis (IPA)

mRNA sequencing was performed in biological triplicate in A549 *STAT1* KD, *VILMIR* KD, and control cell lines treated with mock or either 1 ng/mL or 10 ng/mL human IFN-β for 6 hours. A separate experiment was performed in A549 with mock or IAV CA/04/09 H1N1 MOI 0.1 infection for 18 hours. For each experiment, total RNA was isolated from cells following the TRIzol isolation method (Invitrogen). All samples were quantified and assayed to confirm a minimum RNA integrity number of at least 9.4 using an Agilent Bioanalyzer or TapeStation 4150. Next, 500 ng of total RNA per sample underwent mRNA capture and was then fragmented at 94°C for 6 min. Sequencing libraries were prepared according to the manufacturer’s protocol using 11 cycles of final amplification (KAPA mRNA HyperPrep Kit, catalog no. KK8580 and KAPA UDI Adapter Kit, catalog no. KK8727). Libraries underwent QC prior to sequencing using an Agilent Bioanalyzer or TapeStation 4150. Next-generation sequencing was performed on an Illumina NextSeq500 (75 bp paired end) or Complete Genomics DNBSEQ-G400C (150 bp paired end) to a targeted depth of ~20 million reads per sample.

Illumina or Complete Genomics RNA-seq reads were mapped against the Hg38 using STAR version 2.7.9 a (29). Custom STAR parameters were set as follows: limitOutSAMoneReadBytes: 1,000,000, outSAMprimaryFlag: AllBestScore, outFilterType: BySJout, alignSJoverhangMin: 8, alignSJDBoverhangMin: 3, outFilterMismatchNmax: 999, alignIntronMin: 20, alignIntronMax: 1,000,000, alignMatesGapMax: 1,000,000, outFilterMultimapNmax: 20; otherwise, default STAR parameters were used. Following read mapping, a count matrix was generated from the STAR results using R. Genes were removed from the matrix if they did not have at least 30 reads in a minimum of three samples from either the knockdown or control group. Counts were normalized using the TMM normalization method via the calcNormFactors function in edgeR version 3.40.2 (35).

To conduct our differential gene expression analysis, we utilized the limma-trend approach from Limma version 3.54.2 (31). For the IFN-β treatment, our analysis probed the differential gene expression of each of the two interferon treatments for each gene knockdown (including the GFP control) against their respective mock treatments, resulting in a total of eight contrasts. We then contrasted the differential expression results of each knockdown (STAT1g1/1 ng IFN-β vs STAT1g1/Mock, VILMIRg1/1 ng IFN-β vs VILMIRg1/Mock, etc.) against that of the corresponding control (Ctrl/1 ng IFN-β vs Ctrl/Mock, Ctrl/10 ng IFN-β vs Ctrl/Mock), resulting in a total of six additional contrasts. The H1N1 infection was analyzed similarly. Significantly differentially expressed genes were defined using two different threshold criteria. For our first cutoff, genes were considered differentially expressed in a given contrast if their unadjusted *P*-value was less than 0.05 with no fold change requirement. Our second, stricter threshold required an adjusted *P*-value of less than 0.05 with a minimum fold change of 1.25 (absolute value of log2 fold change >~0.32) for a gene to be considered differentially expressed. The results of our differential gene expression analysis were then plotted using the ComplexHeatmap R package version 2.14.0 (48). Locally estimated scatterplot smoothing curves of each contrast were plotted using the ggplot2 R package version 3.4.3 (49).

Pathway enrichment analysis and disease and function enrichment analysis were generated using QIAGEN Ingenuity Pathway Analysis (IPA) (50). A raw *P*-value cutoff of <0.05 was used to define genes with significant expression changes after *VILMIR* KD in each cell line and IFN-β treatment or H1N1 infection. Canonical pathways analysis identified the pathways from the QIAGEN IPA library of canonical pathways that were most significant to the data set, and the Diseases & Functions Analysis identified the biological functions and/or diseases that were most significant from the data set. Differentially expressed genes from the data set that met the *P*-value cutoff of 0.05 (−log10 *P*-value 1.3) and were associated with a canonical pathway or function and/or disease in the QIAGEN Knowledge Base were considered for the pathway and disease/function analysis. A right-tailed Fisher’s exact test was used to calculate a *P*-value determining the probability that the association between the genes in the data set and the canonical pathways or functions/disease is explained by chance alone.

## RESULTS

### Human lncRNA *VILMIR* is consistently upregulated after influenza infection by a compendium of bulk RNA-seq analysis

**Fig 1:**
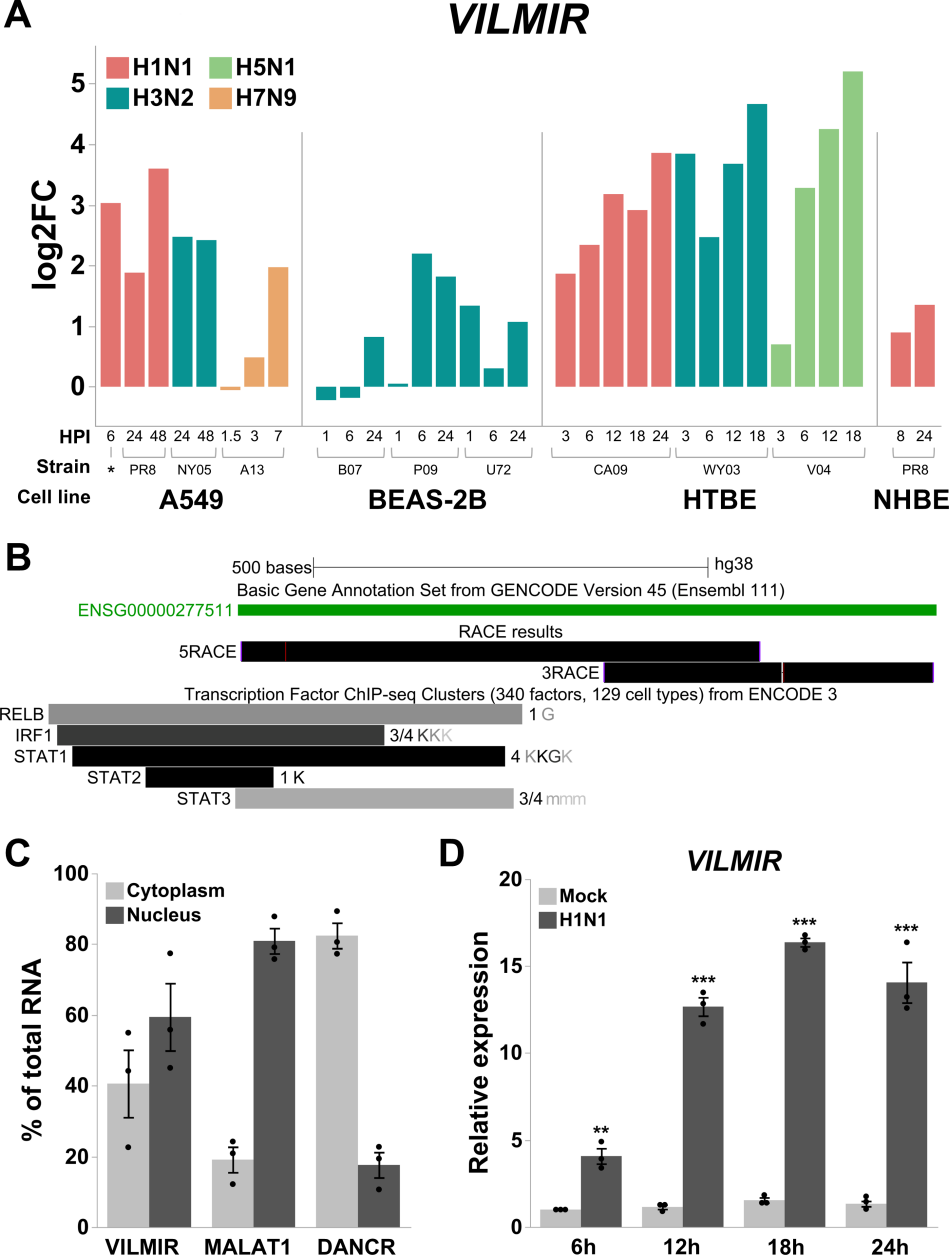
*VILMIR* identified as an lncRNA upregulated after influenza A infection in human epithelial cells. (A) Temporal expression changes of lncRNA *VILMIR* in four human epithelial cell lines infected with various IAV strains in 121 RNA-seq samples (see Materials and Methods for data collection). The *x*-axis indicates human epithelial cell line, IAV strain, and hours post-infection (HPI). The bars are colored by IAV subtype according to the legend. Detailed strain information and MOI are displayed in Table 1. The *y*-axis represents log2FC of *VILMIR* between infected samples and mock-treated control samples in each experiment. (B) UCSC Genome Browser view of Sanger sequencing results of the 5´ and 3´ end sequences of lncRNA *VILMIR* determined by 5´ and 3´ RACE (black RACE results track) aligned to the Hg38 GENCODE V45 annotation (ENSG00000277511 in green). In addition, Transcription Factor ChIP-seq Clusters from ENCODE 3 showing RELB, IRF1, STAT1, STAT2, and STAT3 binding sites near the 5´ end. Gray boxes represent the peak cluster of each transcription factor, and the darkness of the box is proportional to the maximum signal strength observed in any cell type. Labels to the right of the box represent cell types contributing to that cluster, with the darkness of the letters proportional to the signal strength in that cell line (ENCODE cell types are abbreviated as K = K562; G = GM12878; m = MCF_10A, MM.1S, mammary epithelial cell, medulloblastoma, myotube) (http://genome.ucsc.edu). (C) Expression of *VILMIR,* a nuclear control (*MALAT1*), and a cytoplasmic control (*DANCR*) was analyzed by RT-qPCR in cytoplasmic and nuclear fractions from A549 cells. Data were normalized to the total RNA fraction and expressed as % of total RNA (means ± SE; *n* = 3). (D) Relative expression of *VILMIR* was determined by RT-qPCR after IAV H1N1 CA/04/09 MOI 0.1 infection in A549 cells at the indicated time points and normalized to the mean of mock-treated cells at 6 hours. Data were normalized to 18S using the ΔΔCt method and expressed as means ± SE with individual replicates shown (*n* = 3). *P < 0.05, **P < 0.01, ***P < 0.001 (Student’s *t*-test of H1N1 vs mock at each time point).

We started with identifying lncRNAs consistently differentially expressed during human influenza infections. To do so, we first compiled a large-scale RNA-seq compendium of influenza infections in four human epithelial cell lines including primary and immortalized cell lines and 10 different IAV strains covering both seasonal IAV subtypes (H1N1 and H3N2) and highly pathogenic avian IAV viruses (H5N1 and H7N9), totaling 121 RNA-seq samples, summarized in Table 1 (22–28). To select lncRNAs that were more relevant to IAV infection, we searched for genes that were consistently differentially expressed across different infection conditions (described in Materials and Methods). Briefly, we searched for genes that (i) were consistently detected across each of the human epithelial cell lines, (ii) consistently exhibited large expression changes when the overall host response peaked during infection, and (iii) exhibited significant expression changes early after infection. Applying these criteria to all genes, we obtained a list of 15 candidate ncRNA genes: 13 lncRNAs, 1 small nucleolar RNA (snoRNA), and 1 vault RNA, as well as 98 protein-coding genes including those well known to be involved in influenza infection such as *ISG15*, *IRF7*, and *MX1*, validating our selection strategy. Here, we report one of the candidate lncRNAs that met the criteria described above, ENSG00000277511 or *VILMIR*, and showed robust upregulation after influenza infection across all RNA-seq conditions (Fig. 1A), as well as after other respiratory viral infections and IFN-β treatment, detailed below. We reasoned that this robust transcriptional response after viral infection indicated that *VILMIR* may play an important functional role during viral infection.

*VILMIR* is a long noncoding RNA transcript computationally annotated in Ensembl release 77 2014 (51). It is a single exon gene located on chromosome 17, 14 kilobases upstream of its closest neighboring protein-coding gene, *RHOT1,* and 74.083 kilobases downstream of its next closest protein-coding neighbor, *LRRC37B*. As the annotations of lncRNAs tend to be less accurate than those of protein-coding genes, we first validated the accuracy of the existing *VILMIR* annotation for its transcript boundaries, novel isoforms, and coding potential. We used 5´ and 3´ RACE in the human epithelial cell line A549 to confirm the Hg38 GENCODE V45 annotation of this gene (4). 5´ and 3´ RACE PCR fragments were Sanger sequenced and aligned to the GENCODE annotation in UCSC Genome Browser (52, 53). The analysis of 5´ and 3´ RACE products revealed that lncRNA *VILMIR* matches closely with the Hg38 GENCODE V45 annotation with a slightly shorter transcript of 877 nucleotides instead of 885, with no indication of additional novel isoforms (Fig. 1B).

In addition to sequence, determining subcellular localization of lncRNAs is also important in investigating function, as lncRNAs have been found to regulate transcription in the nucleus or translation and signaling in the cytoplasm, among other functions (54). We performed RNA fractionation in A549 cells and found that *VILMIR* was distributed in both nuclear and cytoplasmic fractions, suggesting it could function in or cycle between both compartments (Fig. 1C). As *VILMIR* was distributed in the cytoplasm, we investigated its coding potential by using the coding potential prediction tool, CPPRED (55). The tool used a support vector machine trained on known human coding and noncoding genes and reported only 1.07% probability that *VILMIR* had coding potential. In addition, since ribosome profiling (Ribo-seq) can experimentally uncover open reading frames (ORFs) within lncRNAs, we searched a recent publication cataloguing 7,264 ORFs found in lncRNAs and untranslated regions of protein-coding genes in Ribo-seq data sets from seven different publications (56), and found that *VILMIR* was not one of the identified lncRNAs that contains an ORF. These results indicate that *VILMIR* likely does not code for proteins.

Finally, as we relied on public RNA-seq data to prioritize *VILMIR* as a gene of interest, we sought to experimentally validate the response of *VILMIR* to influenza infection using RT-qPCR. We infected A549 epithelial cells with the commonly circulating H1N1 CA/09 IAV strain at an MOI of 0.1 and collected total RNA at various time points after infection to analyze expression. Our RT-qPCR results confirmed that *VILMIR* was significantly upregulated after H1N1 IAV infection at the earliest collected time point of 6 hours post-infection and increased up to 16-fold at 18 hours post-infection (Fig. 1D). Expression of *VILMIR* began to plateau and drop at 24 hours post-infection, which is also when cytopathic effects on the cells were observed, indicating this drop is likely due to cell death.

### *VILMIR* expression is upregulated across multiple respiratory viral infections

**Fig 2:**
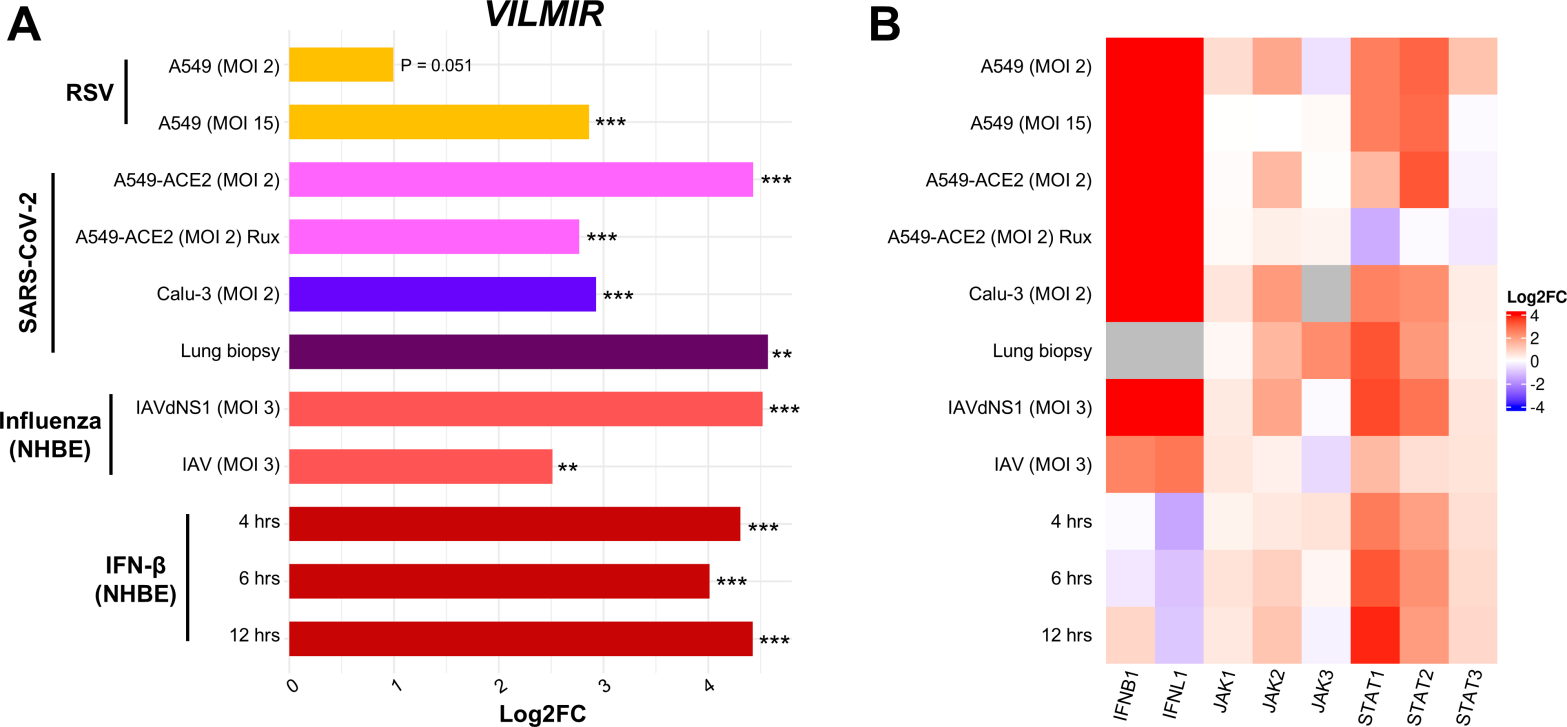
*VILMIR* is upregulated in response to RSV, SARS-CoV-2, influenza A viruses, and interferon-β. (A) Horizontal barplot showing log2FC between infected or treated samples and their corresponding mock-treated control samples in each condition as indicated by the label on the left. The *y*-axis represents experimental conditions of RNA-seq samples from GSE147507 (33, 34). The color of the bars represents the virus and host cell line for each set of samples. From top to bottom: yellow color for samples treated with RSV A2 strain; pink, blue, and purple for samples infected with SARS-CoV-2 in the labeled cell types; salmon for samples treated with influenza A/Puerto Rico/8/1934 WT or an NS1-deleted strain (IAVdNS1); and red for samples treated with human IFN-β. (B) Heatmap showing the expression changes (log2FC) of selected *IFN*, *JAK,* and *STAT* genes in the same samples when compared to matched controls. Red: upregulation; blue: downregulation; gray: insufficient numbers of RNA-seq reads to produce a measure. **P < 0.01, ***P < 0.001 (unadjusted *P*-value of infected or treated vs mock).

To investigate if the upregulation of *VILMIR* was unique to influenza infection, we collected additional RNA-seq data (33, 34) and calculated *VILMIR* expression changes during several respiratory viral infections as well as IFN-β treatment in various human epithelial cell types including primary and immortalized cell lines. We observed that *VILMIR* was upregulated in infected or treated samples compared to mock-treated samples after RSV infection, SARS-CoV-2 infection, additional influenza infections, and IFN-β treatment (Fig. 2A). As expected, the expression levels of genes in canonical interferon signaling such as interferon and JAK*/*STAT genes were also upregulated in these conditions (Fig. 2B).

Interestingly, *VILMIR* upregulation decreased when host interferon responses were suppressed. For example, the upregulation of *VILMIR* decreased significantly ( (log2FC of 2 or a decrease of fourfold, unadjusted *P*-value <0.001) in normal human bronchial epithelial cells infected with wild-type IAV (log2FC 2.5, unadj. *P*-value <0.01) compared to cells infected with a mutant IAV lacking the NS1 protein (dNS1) (log2FC 4.5, unadj. *P*-value <0.001), a known inhibitor of the host interferon response (57) (Fig. 2A). This also aligned with decreased upregulation of multiple IFN signaling genes in the wild-type IAV-infected cells, such as *IFNB1*, *IFNL1*, *STAT1*, and *STAT2* (Fig. 2B). However, as the infection kinetics of these two viruses were not analyzed in this study, it is not clear whether the decrease in *VILMIR* expression was due solely to the presence of NS1 or from differences in the viral infection and subsequent replication. In SARS-CoV-2 infection of ACE2-modified A549 cells, the upregulation of *VILMIR* also decreased significantly (log2FC of 1.7 or a decrease of 3.2-fold, unadj. *P*-value <0.001) in infected cells pretreated with ruxolitinib (log2FC 2.7, unadj. *P*-value <0.001), a JAK1 and JAK2 inhibitor, compared to infected cells not pretreated with ruxolitinib (log2FC 4.4, unadj. *P*-value <0.001) (Fig. 2A). As *VILMIR* upregulation decreased after each of these conditions but was not completely abolished, these results suggest that *VILMIR* may be regulated by multiple pathways.

### *VILMIR* is an interferon-stimulated gene and is associated with a type I IFN response

**Fig 3:**
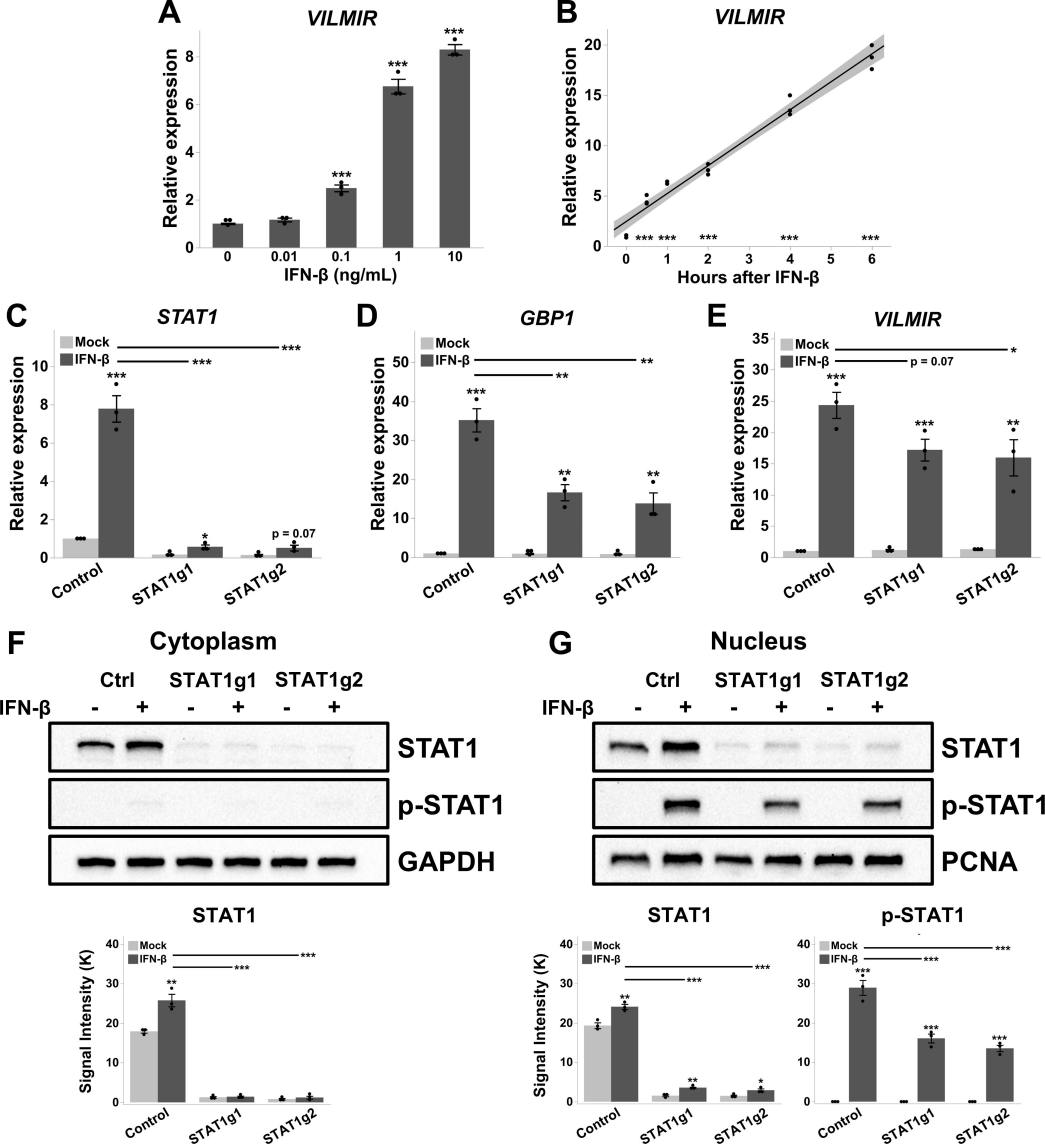
*VILMIR* expression after IFN-β treatment in A549 cells. (A) Relative expression of *VILMIR* determined by RT-qPCR after treatment with the indicated concentrations of 0 ng/mL–10 ng/mL human IFN-β at 6 hours post-treatment in A549 cells (*n* = 3). (B) Relative expression of *VILMIR* determined by RT-qPCR after treatment with 10 ng/mL human IFN-β at 0, 0.5, 1, 2, 4, and 6 hours post-treatment in A549 cells. Individual replicates at each time point were plotted along with a regression line (black) and 95% CI (gray shadow) (*n* = 3, one-way analysis of variance *post hoc* Dunnett). (C–E) A549 cells expressing dCas9-KRAB were transduced with two guide RNAs targeting *STAT1* (STAT1g1 and STAT1g2) along with a control gRNA. The cell lines were treated with mock or 10 ng/mL human IFN-β for 6 hours. Relative expression of (C) *STAT1*, (D) *GBP1*, and (E) *VILMIR* in the gRNA lines was determined by RT-qPCR and normalized to the mock-treated control cell line. The average of three independent experiments is shown. Unless otherwise stated, RT-qPCR data were normalized to GAPDH using the ΔΔCt method and expressed as means ± SE (*n* = 3). *P < 0.05, **P < 0.01, ***P < 0.001 (Student’s *t*-test vs 0 or the mock for each unless indicated). (F and G) In the same samples from panels C–E, STAT1 and p-STAT1 proteins were analyzed by Western blot in (F) cytoplasmic and (G) nuclear protein lysate. GAPDH and PCNA were used as loading controls for cytoplasmic and nuclear lysate, respectively. A representative blot of three independent experiments is displayed for each. Below each blot, densitometry analysis of STAT1 and p-STAT1 is shown by averaging the signal intensity of the target protein from three independent experiments. The target protein was normalized to the loading control on each blot. In the cytoplasmic lysate, p-STAT1 signal was too low to detect accurately. *P < 0.05, **P < 0.01, ***P < 0.001 (Student’s *t*-test)

The observations that *VILMIR* was induced after several respiratory viral infections as well as interferon treatment (Fig. 2A) indicated that *VILMIR* was likely an ISG. To evaluate whether the upregulation of *VILMIR* to interferon was dependent on the dose of interferon treatment, we treated A549 cells with increasing concentrations of human IFN-β from 0 to 10 ng/mL for 6 hours and measured *VILMIR* expression by RT-qPCR. We observed a dose response of *VILMIR* with relative expression increasing as the IFN-β concentration increased (Fig. 3A).

We also found that *VILMIR* expression was significantly correlated with hours of IFN-β treatment, showing an increase in expression over a 6 hour time course and an immediate response to interferon within 30 min (Fig. 3B). In addition, using Transcription Factor ChIP-seq Clusters from ENCODE 3 (58, 59), we found evidence for RELB (subunit of NF-κB), IRF1, STAT1, STAT2, and STAT3 binding sites near the 5´ end of *VILMIR* within several human cell lines (Fig. 1B), further supporting our hypothesis that this gene may be a novel ISG.

To determine whether lncRNA *VILMIR* is directly induced by the canonical JAK/STAT signaling pathway or an independent pathway, the expression of *VILMIR* was analyzed by RT-qPCR after the knockdown of *STAT1* using a CRISPR interference system. Briefly, A549 cells expressing dCas9-KRAB and GFP were transduced with two different gRNAs targeting *STAT1* along with a negative control gRNA targeting GFP expression and treated with human IFN-β. As expected, in the negative control gRNA cell line, *STAT1* was upregulated after IFN-β treatment, whereas the STAT1g1 and STAT1g2 cell lines showed about a 93% knockdown of *STAT1* gene expression in the IFN-treated samples compared to the control cell line (Fig. 3C). Successful knockdown of *STAT1* was also confirmed by the inhibition of known ISGs, such as *GBP1*, which had about a 53 and 60% decrease in expression compared to the control (Fig. 3D).

Interestingly, there was a 29 and 34% decrease in *VILMIR* expression in the STAT1g1 and STAT1g2 cell lines, respectively; however, this was not significant for STAT1g1 (Fig. 3E). This suggests that there may be some regulation of *VILMIR* expression by STAT1; however, expression was not completely abolished. As the STAT1 protein gets phosphorylated and translocated to the nucleus upon binding of type I IFNs to induce transcription of ISGs (60), we determined whether upregulation of *VILMIR* could be due to residual phosphorylated STAT1 (p-STAT1) in the *STAT1* KD cell lines by Western blot analysis. Similarly to the RT-qPCR results, STAT1 protein was reduced between 85 and 95% in the KD cell lines compared to the control according to densitometry analysis in both cytoplasmic and nuclear compartments (Fig. 3F through G). However, p-STAT1 was still present in the nucleus and was only reduced by 44 and 53% in the STAT1g1 and STAT1g2 KD lines compared to the control (Fig. 3F through G). Therefore, it is possible that residual STAT1 protein in the KD lines was still able to upregulate transcription of certain ISGs, including *VILMIR*.

**Fig 4:**
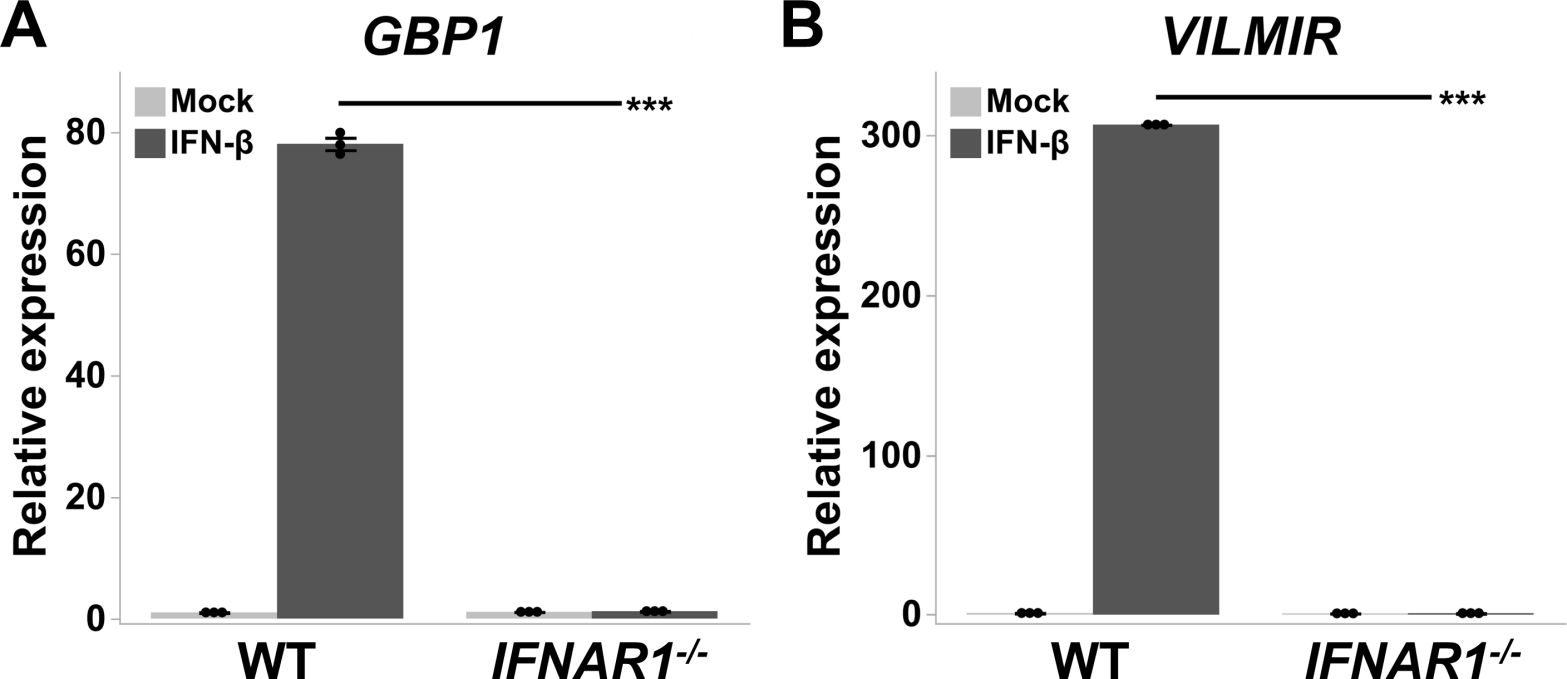
*VILMIR* expression is abolished after IFNAR1 knockout in Huh7 cells. Huh7 WT and *IFNAR1* knockout (*IFNAR1^-/-^*) cell lines were treated with mock or 10 ng/mL human IFN-β for 6 hours. Relative expression of (A) *GBP1* and (B) *VILMIR* was determined by RT-qPCR and normalized to the mean of the mock-treated WT cell line. Data were normalized to GAPDH using the ΔΔCt method and expressed as means ± SE (*n* = 3). ***P < 0.001 (Student’s *t*-test).

To provide more conclusive evidence that *VILMIR* expression is regulated by the type I IFN response, *VILMIR* expression was analyzed after KO of the IFNAR1. Type I IFN binding to IFNAR1 and IFNAR2 activates the JAK/STAT signaling cascade, which results in upregulation of ISGs during the interferon response (60). As the cellular components of the canonical type I IFN signaling pathway are widely expressed by most cell types (61), we obtained Huh7 WT and *IFNAR1* KO cells (36), an epithelial-like liver cell line, and treated these with mock or IFN-β. As expected, in the Huh7 WT cells, expression of both a canonical ISG, *GBP1*, as well as *VILMIR* was upregulated after IFN-β treatment (Fig. 4). However, after KO of IFNAR1, both *GBP1* and *VILMIR* expressions were abolished (Fig. 4). These results support our hypothesis that *VILMIR* is a novel ISG and is strongly associated with the type I IFN response.

### *VILMIR* is upregulated in immune cell types during SARS-CoV-2 infection and interferon-β treatment

**Fig 5:**
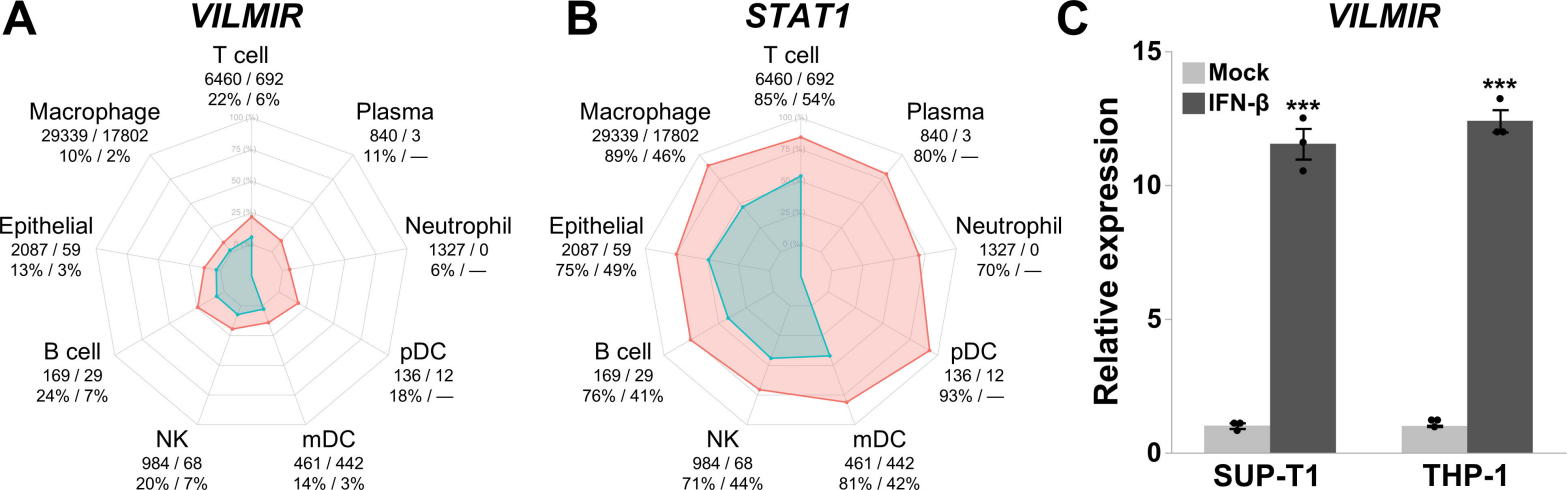
*VILMIR* is expressed in immune cell types after SARS-CoV-2 infection and IFN-β treatment. (A) Overall percentage of cells expressing *VILMIR* from each cell type in SARS-CoV-2-infected and uninfected BALF from GSE145926 (44). Red points represent the overall percentage by combining cells of nine SARS-CoV-2-infected samples. Blue points represent the overall percentage by combining cells of three uninfected control samples. Each cell type is labeled with the total number of cells across infected and control samples, as well as the percentage of infected cells to control cells that express *VILMIR* (infected/control). A dash is indicated for cell types that had low relative abundance in control samples (see methods). Cell types are abbreviated as NK = natural killer; mDC = monocyte-derived dendritic cells; and pDC = plasmacytoid dendritic cells. Sample-specific cell counts for each cell type can be found in Table S1. (B) Similarly, as in A, the overall percentage of cells expressing *STAT1* from each cell type in SARS-CoV-2-infected and uninfected BALF. (C) Relative expression of *VILMIR* determined by RT-qPCR after treatment with 10 ng/mL human IFN-β for 6 hours in the human cell lines, SUP-T1 T cells and THP-1 monocytes. All RT-qPCR data were normalized to GAPDH using the ΔΔCt method and expressed as means ± SE (*n* = 3). ***P < 0.001 (Student’s *t*-test vs the mock for each cell type).

As our analysis of *VILMIR* was focused primarily on human epithelial cells, we were interested in whether *VILMIR* may be expressed in immune cell types. We obtained published single-cell RNA-seq data from BALF samples from COVID-19 patients and uninfected control samples (44). After confirming that *VILMIR* was detected in SARS-CoV-2-infected samples, we adopted the same cell type annotation as in reference 44 to investigate which cell types expressed *VILMIR*. We focused on a subset of nine annotated cell types that were relatively abundant in these samples (see Materials and Methods): macrophages, T cells, epithelial cells, natural killer cells, neutrophils, monocyte-derived dendritic cells (mDCs), plasma cells, B cells, and plasmacytoid dendritic cells (pDCs). Since for multiple cell types, the number of cells in individual samples was zero or very low, for each cell type, we calculated an overall percentage of cells where expression of *VILMIR* was detected by combining cells from individual samples, separately for infected and control samples (Fig. 5A). Excluding three cell types (plasma, neutrophil, and pDC) which had extremely low numbers of cells in the uninfected control samples (15 cells in total for these three cell types, Table S1), we observed an increase in the overall percentage of cells expressing *VILMIR* in the SARS-CoV-2-infected samples when compared to uninfected control samples in the remaining six cell types (Fig. 5A). We also performed the same calculation for *STAT1,* representing the canonical innate immune response and observed similar trends as expected (Fig. 5B).

For T cells and macrophages, the top two most abundant cell types that also had a sufficient number of cells in individual samples, we performed statistical testing and found the changes in percentage of cells expressing *VILMIR* to be statistically significant between the infected samples and the uninfected controls (Wilcoxon rank sum exact test unadjusted *P*-value <0.01 for both).

To determine if upregulation of *VILMIR* could be related to the interferon response in T cells and macrophages, we treated the human T cell line SUP-T1 and the monocyte cell line THP-1 *in vitro* with 10 ng/mL human IFN-β for 6 hours. As expected, we observed significant upregulation of *VILMIR* in both cell lines after IFN-β treatment, confirming IFN-induced expression of *VILMIR* in human T cells and monocytes (Fig. 5C). Overall, these results indicate that *VILMIR* can be activated in response to SARS-CoV-2 infection and IFN treatment in at least two immune cell types, in addition to epithelial cells as described above.

### Knockdown of *VILMIR* dampens the host response to interferon-β treatment in A549 epithelial cells

**Fig 6:**
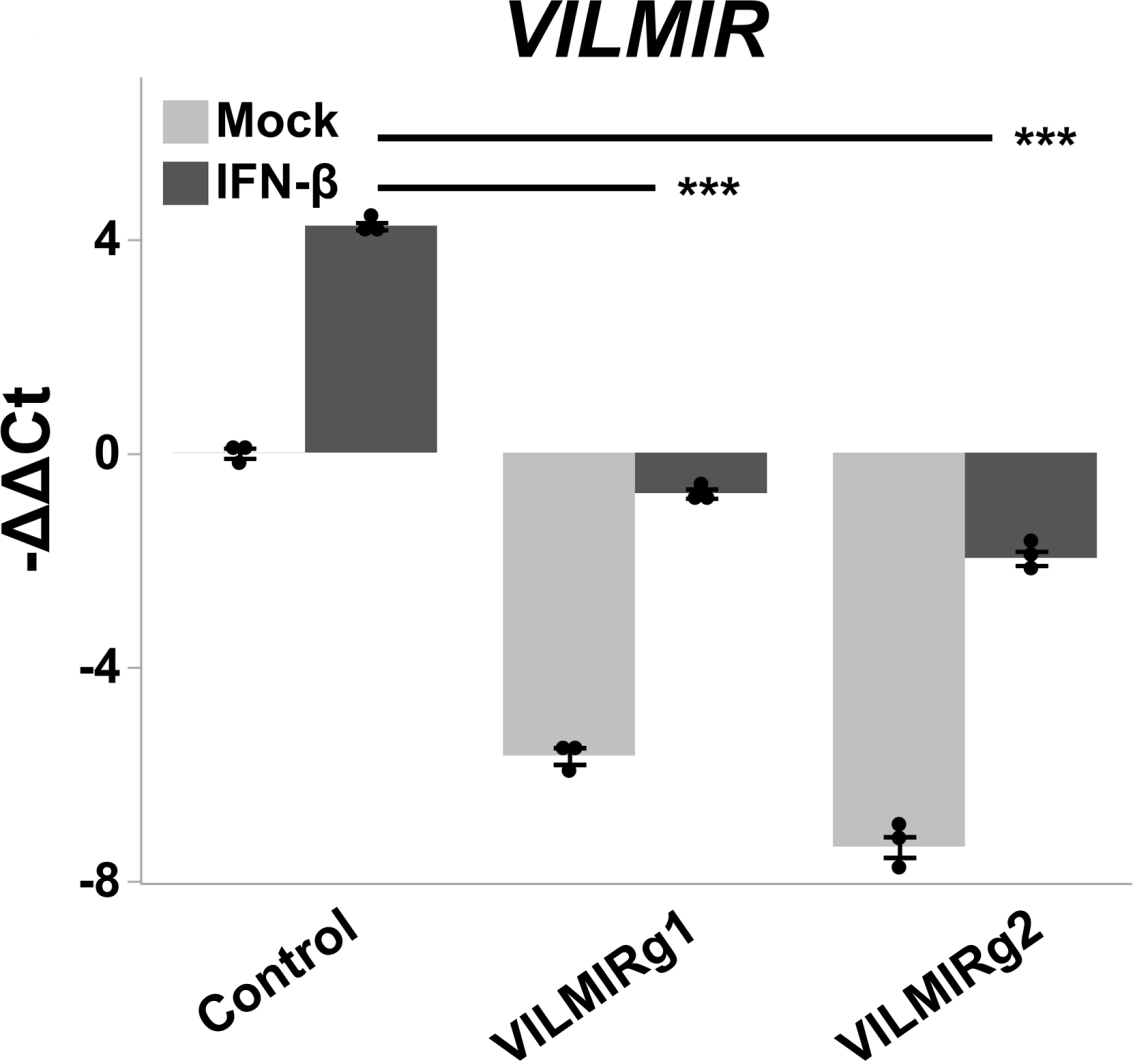
Knockdown efficiency of *VILMIR* expression after IFN-β treatment using CRISPRi. A549 cells expressing dCas9-KRAB were transduced with two gRNAs targeting *VILMIR* (VILMIRg1 and VILMIRg2) along with a control gRNA targeting GFP expression (Control) and treated with mock or 10 ng/mL human IFN-β for 6 hours. Relative expression of *VILMIR* was determined by RT-qPCR and normalized to the mean of the mock-treated control cell line. Data were normalized to GAPDH using the ΔΔCt method and expressed as means ± SE (*n* = 3). ***P < 0.001 (Student’s *t*-test).

To investigate the functional role of *VILMIR* during the host interferon response, we repressed *VILMIR* expression using CRISPRi interference, as described above. A549 cells expressing dCas9-KRAB and GFP were transduced with two gRNAs targeting the 5´ end of *VILMIR* along with a negative control gRNA targeting GFP expression and treated with or without two separate concentrations of human IFN-β. Compared to the control cell line with a gRNA targeting GFP, we observed about 96% KD of *VILMIR* expression (Fig. 6). We then performed RNA-seq analysis to identify the impact of *VILMIR* KD on the host transcriptional response to IFN-β treatment. In addition, a *STAT1* KD cell line (STAT1g1) was included for comparison, as *STAT1* is well-known to impact the host IFN response and served as a positive control for impact on host transcriptional response.

**Fig 7:**
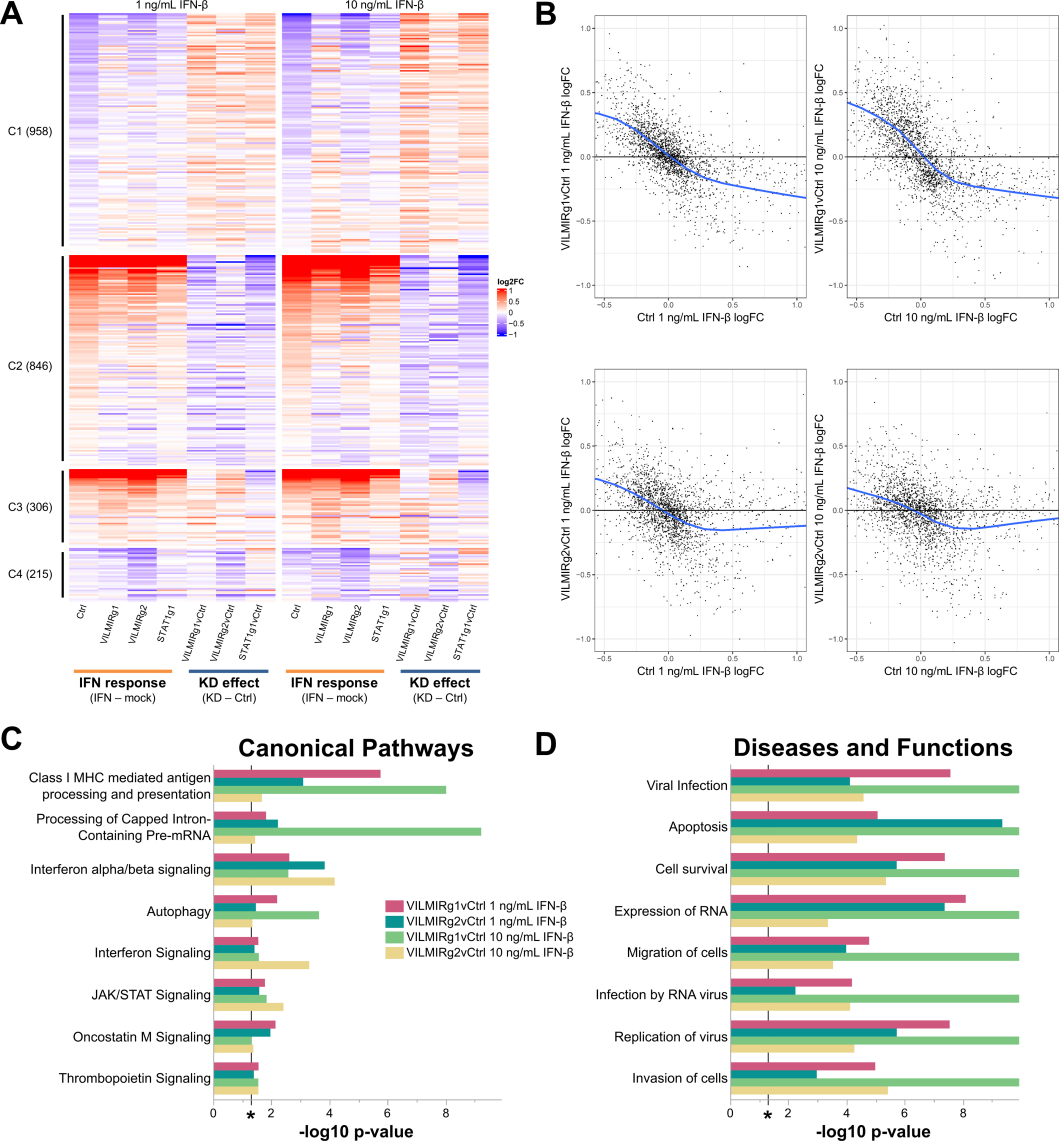
Transcriptional and pathway analyses of the effect of *VILMIR* knockdown on IFN-β treatment in A549 cells. (A) Heatmap overview of the RNA-seq analysis of *VILMIR* knockdown (VILMIRg1 and VILMIRg2), *STAT1* knockdown (STAT1g1), or control (Ctrl) A549 gRNA cell lines that were treated with mock or either 1 ng/mL or 10 ng/mL human IFN-β for 6 hours (*n* = 3). The heatmap displays 2,325 human genes that exhibited significant changes in their responses to IFN-β treatment after *VILMIR* KD in at least one KD cell line treated with one of two doses of IFN (raw *P*-value <0.05). Rows are genes and columns are conditions and comparisons. As shown by the labels at the bottom, the log2FC after IFN-β treatment in each cell line was first calculated (“IFN response”), and then the “KD effect” was calculated by comparing the “IFN response” log2FC of each KD line to the “IFN response” log2FC of the control cell line. Red color indicates positive log2FC value (i.e., upregulation) in columns above the label “IFN response,” or higher log2FC values in knockdown cells compared to that of control cells in columns above the label “KD effect.” The blue color indicates negative log2FC value (i.e., downregulation) in columns above the label “IFN response,” or lower log2FC values in knockdown cells compared to that of control cells in columns above the label “KD effect.” Genes are grouped into four clusters based on their expression patterns across conditions/comparisons (columns): C1. The expression of 958 genes was downregulated by IFN treatment as shown by the “Ctrl” column, but the magnitude of downregulation was decreased in *VILMIR* knockdown cells. C2. The expression of 846 genes was upregulated by IFN treatment as shown by the “Ctrl” column, but the magnitude of upregulation was decreased in *VILMIR* knockdown cells. C3. The expression of 306 genes was upregulated by IFN treatment as shown by the “Ctrl” column, but the magnitude of upregulation was increased in *VILMIR* knockdown cells. C4. The expression of 215 genes was downregulated by IFN treatment as shown by the “Ctrl” column, but the magnitude of downregulation was increased in *VILMIR* knockdown cells. The full list of genes and log2FC values is available in Table S2. (B) Scatterplots of the “KD effect,” i.e., the differences between log2FC values in each KD line to the log2FC values in control cell line, vs the “IFN response” log2FC values in control cell line for the same 2,325 genes shown in (A). The blue line represents the locally estimated scatterplot smoothing (LOESS) curve, and the black line at 0 on the *y*-axis represents if no change was observed after KD. Cutoffs were applied on the *y*-axis (−1.0–1.0) and *x*-axis (−0.5–1.0) for the data points. The full plots and mean squared error (MSE) can be seen in Fig. S2. (C) QIAGEN IPA was performed to identify canonical pathways significantly enriched in the DEGs impacted by *VILMIR* KD after 1 ng/mL or 10 ng/mL IFN-β treatment in A549 cells (Tables S3 to S5). Shown are the eight significant pathways shared between both *VILMIR* KD lines and IFN-β treatments. (D) In addition, diseases and biological functions categories significantly enriched in the DEGs were identified for both IFN-β treatments using IPA (cancer categories excluded). Enriched pathways and functional categories that met the raw enrichment *P*-value <0.05 (−log10 *P*-value cutoff of 1.3) using a right-tailed Fisher’s exact test in both *VILMIR* KD lines and were associated with a canonical pathway or function and/or disease in the QIAGEN Knowledge Base were included here (*P < 0.05 included as reference). For visualization, a −log10 *P*-value cutoff of 9.9 was applied to the *x*-axis of panel D. The plot in D represents a subset of enriched functional categories found in Table S6.

We first examined the overall effects of *VILMIR* KD on the expression responses to IFN-β treatment using relatively relaxed criteria for differential expression analysis, i.e., raw *P*-value <0.05, to investigate the potentially broad regulatory roles of *VILMIR*. Using this criterion, we identified 2,325 genes that showed altered expression changes to IFN-β treatment after *VILMIR* KD in at least one KD cell line treated with one of two doses of IFN-β (Fig. 7A; Table S2). However, the average magnitude of expression changes was relatively small, with gene expression changing between 1.26- and 1.29-fold after *VILMIR* KD compared to the control. While *RHOT1* and *LRRC37B*, the two immediate neighboring protein-coding genes of *VILMIR*, were identified in this list of 2,325 genes, *RHOT1* expression changes were only significantly affected in one out of the four conditions. Independent RT-qPCR analysis also showed the effect of *VILMIR* KD on the *RHOT1* expression changes was not significant (Fig. S1A through C). In comparison, *LRRC37B* expression changes were significantly affected in three out of the four conditions by RNA-seq analysis, and this effect on *LRRC37B* expression changes was also confirmed by RT-qPCR for both *VILMIR* KD lines in the 10 ng/mL IFN-β treatment (Fig. S1D and E). Since the observed magnitude of *VILMIR* KD effect on *LRRC37B* expression change was relatively small (average −ΔΔCt difference of −0.27 or a decrease of 0.83-fold by RT-qPCR) and *LRRC37B* was reported as a membrane receptor in neurons (62), the potential effect of *VILMIR* KD on *LRRC37B* needs to be further investigated.

To model the overall trend of the KD impact on host transcriptional response, a local regression analysis was performed by plotting the log2FC values in each KD line to the log2FC values in the control versus the log2FC values in the control cell line for the 2,325 genes identified in the RNA-seq data. Interestingly, we found that after *VILMIR* KD, the magnitudes of the expression changes induced by IFN-β treatment decreased in general, similarly as in the *STAT1* KD line (Fig. 7B; Fig. S2). Generally, genes that were downregulated by IFN-β treatment also showed downregulation after *VILMIR* KD but with smaller fold changes (Fig. 7A, cluster 1). Likewise, genes that were upregulated by IFN-β treatment also showed upregulation after *VILMIR* KD but with smaller fold changes (Fig. 7A, cluster 2). In total, clusters 1 and 2 represented 78% of the 2,325 genes identified. There were two smaller clusters of genes that were upregulated or downregulated with higher fold changes after *VILMIR* KD (Fig. 7A, clusters 3 and 4, respectively), representing 22% of the total genes identified. This indicates an overall opposite effect on host response between the KD of *VILMIR* and the IFN-β treatment. As KD of *STAT1* showed a similar trend across the same genes (Fig. 7A), and *STAT1* is known to be a key activator of the IFN response, these results also suggest *VILMIR* might play an activating role in the IFN response.

To identify canonical pathways enriched in the DEGs impacted by *VILMIR* KD, QIAGEN Ingenuity Pathway Analysis (IPA) was performed (50). To account for differences in the dose of IFN-β, the two IFN-β treatments were separated for the pathway analysis. In addition, we only focused on significant pathways that were shared between both KD lines to control for any off-target effects or variation between the KD lines. In the 1 ng/mL IFN-β treatment, 36 canonical pathways were significantly enriched in both KD lines with a raw enrichment *P*-value <0.05 (−log10 *P*-value >1.3, Table S3), while 66 canonical pathways were significantly enriched in both KD lines in the 10 ng/mL IFN-β treatment (Table S4). The longer list of 66 pathways enriched in the 10 ng/mL IFN-β treatment may be due to increased cellular stress caused by the higher dosage of IFN-β (63), as several of these pathways were specifically linked to cell death and survival functions, such as p53 Signaling, 14-3-3-mediated Signaling, and Death Receptor Signaling. When comparing the two doses of IFN-β, there were eight significantly enriched pathways shared between both KD lines and treatments (Fig. 7C; Table S5). Interestingly, these included several interferon-related pathways (Interferon alpha/beta signaling, Interferon Signaling, JAK/STAT Signaling), as well as pathways representing cell-mediated immunity (Class I MHC-mediated antigen processing and presentation) and cytokine signaling (Oncostatin M Signaling). In addition, pathways representing RNA post-transcriptional modification (Processing of Capped Intron-Containing Pre-mRNA), cellular degradation in response to stress (Autophagy), and proliferation mediated through the JAK/STAT pathway (Thrombopoietin Signaling) were significant.

In addition, an enriched disease and biological function comparison was performed for DEGs impacted by *VILMIR* KD using IPA. While 296 diseases and functions classified by IPA were significantly represented in both KD lines and IFN-β treatments with a raw enrichment *P*-value <0.05 (−log10 *P*-value >1.3), this list decreased to 88 when cancer categories were excluded (Table S6). Significantly enriched diseases and functions of potential relevance included viral infection, apoptosis, cell survival, expression of RNA, and infection by RNA virus, among others (Fig. 7D). It was observed that VILMIRg1 had much lower *P*-values than VILMIRg2 in the 10 ng/mL IFN-β treatment (Fig. 7D), which is likely due to a difference in the number of DEGs in VILMIRg1 compared to VILMIRg2 (1,265 and 508 DEGs, respectively).

To examine the more robust expression changes after *VILMIR* knockdown and to mitigate potential off-target effects, we applied additional filtering to the differential expression analysis and obtained a list of 78 genes that showed altered expression changes to IFN-β treatment in the same direction across both KD cell lines by 1.25-fold or greater in at least one IFN-β treatment (Fig. S3). The same observation was made for this subset of genes, with the majority of genes exhibiting smaller fold changes after *VILMIR* KD (Fig. S3, clusters 1 and 2). Again, a very small percentage of genes exhibited larger fold changes after *VILMIR* KD (Fig. S3, clusters 3 and 4). Two ISGs identified in this list of 78 were *IFIT2* and *IFI44L*, which decreased in their responses to IFN-β treatment after *VILMIR* KD. *IFIT2* decreased by twofold and 1.6-fold in the VILMIRg1 and VILMIRg2 KD lines, respectively, after 10 ng/mL IFN-β treatment. Similarly, *IFI44L* decreased by 1.5-fold and 1.8-fold in the VILMIRg1 and VILMIRg2 KD lines, respectively. *IFIT2* and *IFI44L* have previously been identified as ISGs during viral infection (64, 65), supporting our hypothesis that *VILMIR* may regulate the host interferon response. While the overall magnitude of observed changes after *VILMIR* KD was relatively small, these results indicate that *VILMIR* KD can broadly dampen the host response to interferon treatment in A549 epithelial cells.

### *VILMIR* knockdown also reduces the host response to influenza A infection in A549 epithelial cells

To determine the relevance of *VILMIR* in virus-host interactions, we further investigated the role of *VILMIR* during the host response to influenza A infection using a similar workflow as described above. We infected the same A549 *VILMIR* KD lines with IAV CA/09 H1N1 strain at an MOI of 0.1 for 18 hours since *VILMIR* expression was highest at 18 hours in our previous H1N1 infection time course (Fig. 1D) and then performed RNA-seq analysis to identify the impact of *VILMIR* KD on the host transcriptional response to IAV infection.

**Fig 8:**
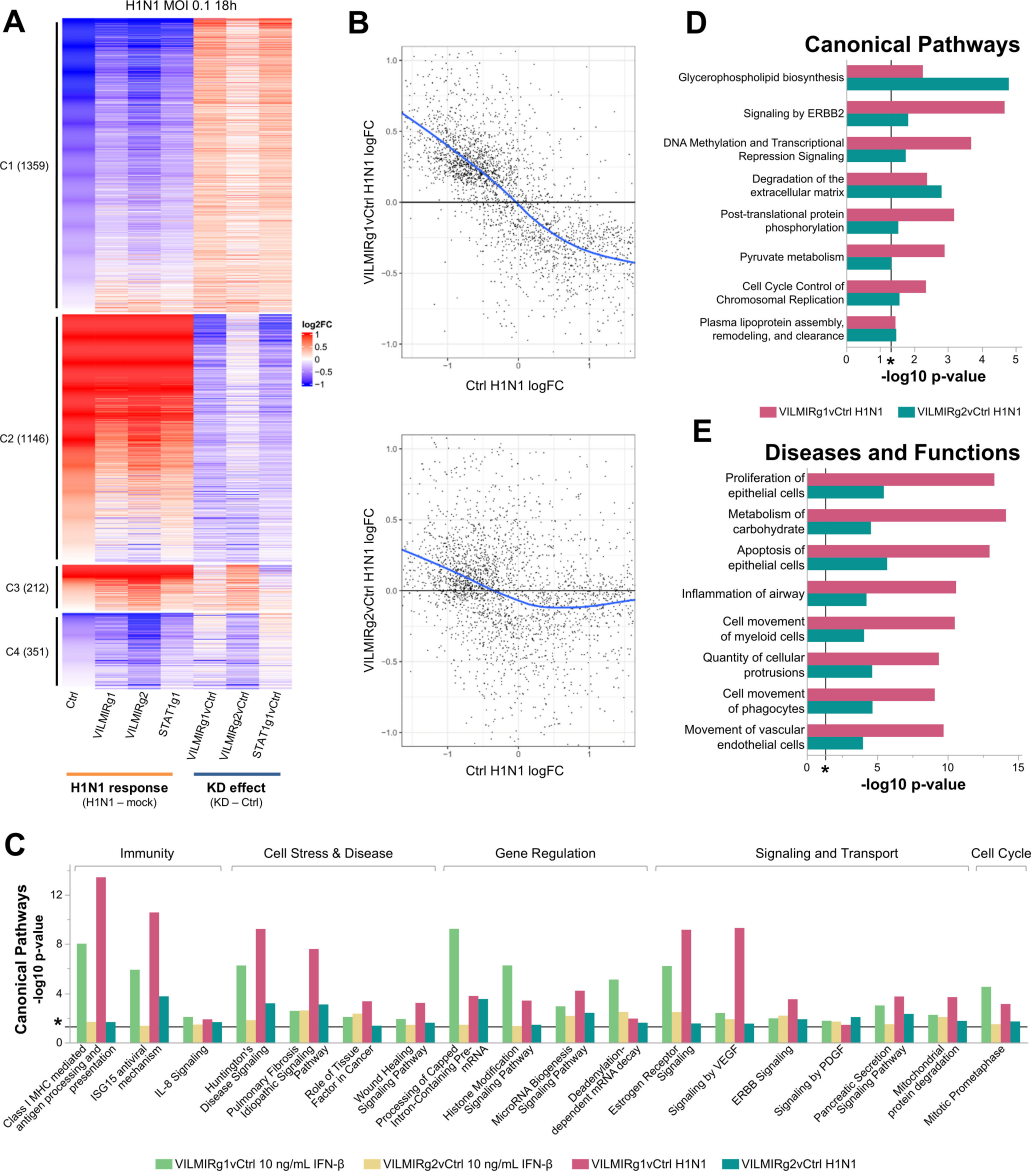
*VILMIR* knockdown reduces the host transcriptional response after IAV H1N1 infection in A549 cells. (A) Heatmap overview of the RNA-seq analysis of *VILMIR* knockdown (VILMIRg1 and VILMIRg2), *STAT1* knockdown (STAT1g1), or control (Ctrl) A549 gRNA cell lines that were treated with mock or IAV H1N1 CA/04/09 MOI 0.1 infection for 18 hours (*n* = 3). The heatmap displays 3,068 human genes that exhibited significant changes in their responses to IAV H1N1 infection after *VILMIR* KD in at least one KD cell line (raw *P*-value <0.05). Rows are genes and columns are conditions and comparisons. As shown by the labels at the bottom, the log2FC after H1N1 infection in each cell line was first calculated (“H1N1 response”), and then the “KD effect” was calculated by comparing the “H1N1 response” log2FC of each KD line to the “H1N1 response” log2FC of the control cell line. Red color indicates positive log2FC value (i.e., upregulation) in columns above the label “H1N1 response,” or higher log2FC values in knockdown cells compared to that of control cells in columns above the label “KD effect.” The blue color indicates negative log2FC value (i.e., downregulation) in columns above the label “H1N1 response,” or lower log2FC values in knockdown cells compared to that of control cells in columns above the label “KD effect.” Genes are grouped into four clusters based on their expression patterns across conditions/comparisons (columns): C1. The expression of 1,359 genes was downregulated by H1N1 infection as shown by the “Ctrl” column, but the magnitude of downregulation was decreased in *VILMIR* knockdown cells. C2. The expression of 1,146 genes was upregulated by H1N1 infection as shown by the “Ctrl” column, but the magnitude of upregulation was decreased in *VILMIR* knockdown cells. C3. The expression of 212 genes was upregulated by H1N1 infection as shown by the “Ctrl” column, but the magnitude of upregulation was increased in *VILMIR* knockdown cells. C4. The expression of 351 genes was downregulated by H1N1 infection as shown by the “Ctrl” column, but the magnitude of downregulation was increased in *VILMIR* knockdown cells. The full list of genes and log2FC values is available in Table S7. (B) Scatterplots of the “KD effect,” i.e., the differences between log2FC values in each KD line to the log2FC values in control cell line, vs the “H1N1 response” log2FC values in control cell line for the same 3,068 genes shown in (A). The blue line represents the locally estimated scatterplot smoothing (LOESS) curve, and the black line at 0 on the *y*-axis represents if no change was observed after KD. Cutoffs were applied on the *y*-axis (−1.0–1.0) and *x*-axis (−1.5–1.5) for the data points. The full plots and mean squared error (MSE) can be seen in Fig. S4. (C) QIAGEN IPA was performed to identify canonical pathways significantly enriched in the DEGs impacted by *VILMIR* KD after both the 10 ng/mL IFN-β treatment and the H1N1 infection in A549 cells (Table S8). The pathways are grouped by common biological categories. (D and E) In addition, IPA was performed to identify (D) canonical pathways or (E) diseases and functions that were significantly enriched after *VILMIR* KD in H1N1 infection alone and none of the IFN-β conditions found in Fig. 7. Enriched pathways and functional categories that met the raw enrichment *P*-value <0.05 (−log10 *P*-value cutoff of 1.3) using a right-tailed Fisher’s exact test in both *VILMIR* KD lines and were associated with a canonical pathway or function and/or disease in the QIAGEN Knowledge Base were included here (*P < 0.05 included as reference). Plots D and E represent a subset of enriched pathways and functional categories found in Tables S12 and S14.

Using the same relaxed criteria as for the IFN-β treatment (raw *P*-value <0.05), we identified 3,068 genes that showed altered expression changes to H1N1 infection after *VILMIR* KD in at least one KD cell line (Fig. 8A; Table S7). Again, the average magnitude of expression changes was small, with gene expression changing between 1.36- and 1.41-fold after *VILMIR* KD compared to the control. As the immediate neighboring protein-coding genes, *RHOT1* and *LRRC37B*, were not identified in this round of RNA-seq, it seems unlikely that *VILMIR* regulates these genes during infection.

Using a local regression analysis, we again found that after *VILMIR* KD, the magnitude of expression changes induced by H1N1 infection decreased in general (Fig. 8B; Fig. S4). In total, 81.6% of the 3,068 genes were downregulated or upregulated with smaller fold changes after *VILMIR* KD compared to the control cell line during H1N1 infection (Fig. 8A, clusters 1 and 2). The remaining 18.4% of genes showed higher fold changes after *VILMIR* KD (Fig. 8A, clusters 3 and 4). As KD of *STAT1* showed a comparable trend across the same DEGs (Fig. 8A), these results suggest that *VILMIR* may play an activating role in the host antiviral response, similarly to *STAT1*.

After applying additional criteria to this analysis to control for potential off-target effects or variation between KD lines, we filtered the 3,068 gene list down to 162 genes that showed altered expression changes to H1N1 infection in the same direction across both KD cell lines by 1.25-fold or greater (Fig. S5). The same observation was made for this subset of genes, with the majority of genes exhibiting smaller fold changes after *VILMIR* KD (Fig. S5, clusters 1 and 2), and a small percentage of genes exhibiting larger fold changes after *VILMIR* KD (Fig. S5, clusters 3 and 4). Two genes identified in this list of 162 were *IFI6* and *CD274*, which decreased in their responses to H1N1 infection after *VILMIR* KD. *IFI6* decreased by 1.6-fold in both KD lines, and *CD274* decreased by 4.5-fold and 1.9-fold in the VILMIRg1 and VILMIRg2 KD lines, respectively. *IFI6* is an ISG previously found to impact production of IAV and SARS-CoV-2 (66). *CD274*, also named programmed death-ligand 1, was found to negatively regulate cytokine expression during IAV infection (67). Therefore, as in the results of *VILMIR* KD after IFN-β treatment, this global trend indicates an opposite effect on the host response between *VILMIR* KD and H1N1 infection, suggesting that *VILMIR* may help to activate the host response to infection.

To predict common regulatory roles of *VILMIR*, we performed another pathway analysis using IPA (50) to identify canonical pathways significantly enriched after *VILMIR* KD in both the DEGs after IFN-β treatment (2,325 genes) and H1N1 infection (3,068 genes). As the H1N1 infection included one treatment as opposed to the two IFN-β doses, we compared each IFN-β dosage to IAV infection separately. In the 10 ng/mL IFN-β and IAV infection comparison, there were 18 pathways that were significantly enriched in both KD lines and treatments with a raw enrichment *P*-value <0.05 (Fig. 8C; Table S8). These pathways included categories such as immunity (ISG15 antiviral mechanism), gene regulation (Histone Modification Signaling Pathway), and signal transduction and molecular transport (Mitochondrial protein degradation). As the 1 ng/mL IFN-β and IAV infection comparison had nine significant pathways that were shared (Table S9), we focused on the longer list of overlapping pathways in the 10 ng/mL treatment. There were only two pathways shared between both IFN-β doses and H1N1 infection, which were Class I MHC-mediated antigen processing and presentation and Processing of Capped Intron-Containing Pre-mRNA. In comparison, when performing a disease and biological function analysis, we found that 68 diseases and functions were significantly enriched in both IFN-β doses and IAV infection, such as apoptosis, viral infection, cell survival, expression of RNA, and infection by RNA virus (Table S10). It was observed that VILMIRg1 KD had lower *P*-values than VILMIRg2 KD during H1N1 infection, which was the same trend observed during IFN-β treatment. This difference is likely due to the number of DEGs in VILMIRg1 compared to VILMIRg2 (2,238 and 1,166 DEGs, respectively). However, despite these differences, the significant pathways that were shared between both KD lines after IFN-β treatment and H1N1 infection indicate that *VILMIR* may play a common regulatory role across the interferon and antiviral responses.

Many viruses, including the influenza virus, antagonize a wide array of host cellular pathways in addition to interferon, such as proliferation, apoptosis, metabolism, and gene expression to create a more favorable environment for viral replication and evade the host immune response (68, 69). Therefore, we were also interested in what pathways were significantly enriched after *VILMIR* KD in only the H1N1 infection. We found that 130 canonical pathways were significantly enriched in both KD lines during H1N1 infection (Table S11), and 32 of these pathways were unique to H1N1 infection and not found in the IFN-β conditions (Fig. 8D; Table S12). These pathways included categories such as phospholipid metabolism (Glycerophospholipid biosynthesis), signaling by receptor tyrosine kinases (Signaling by ERBB2/4), gene expression (DNA Methylation and Transcriptional Repression Signaling), post-translational protein modification (Phosphorylation), and cell cycle regulation (Cell Cycle Control of Chromosomal Replication), all of which have been associated with influenza infection previously (70–74). We also found that 267 diseases and functions classified by IPA were significantly represented in both KD lines, excluding cancer categories (Table S13). Out of these, 44 were unique to H1N1 infection and none of the IFN-β conditions, such as proliferation of epithelial cells, metabolism of carbohydrates, apoptosis of epithelial cells, and inflammation of airway (Fig. 8E; Table S14). While future work is needed to narrow down which pathways are directly downstream of *VILMIR*, these results support our hypothesis that *VILMIR* is an ISG that not only plays an activating role in the interferon response but may also impact broader host responses during respiratory viral infection.

## DISCUSSION

Here, through large-scale RNA-seq analysis, we identified a previously uncharacterized human lncRNA, *VILMIR*, that is significantly upregulated in response to major respiratory viral infections such as influenza, SARS-CoV-2, and RSV in human epithelial cells. *VILMIR* is also upregulated in a dose- and time-dependent manner after human IFN-β treatment and is strongly associated with a type I IFN response. In addition, it responds to SARS-CoV-2 infection and IFN-β treatment in multiple immune cell types. Furthermore, knockdown of *VILMIR* expression broadly dampens the host transcriptional response to both IFN-β treatment and IAV H1N1 infection in A549 cells. Similarly, a recent publication characterized a different lncRNA, *USP30-AS1*, by identifying that *USP30-AS1* is an ISG upregulated by multiple IAV subtypes and is important in modulating the inflammatory response during IAV infection (75). This study, as well as ours, highlights the importance of investigating lncRNAs within the antiviral response in order to identify potentially novel therapeutic targets for modulating the host response to infections.

Interestingly, as shown in RNA-seq analysis, *VILMIR* expression was reduced but not abolished in conditions with suppressed host IFN responses, such as after treatment with ruxolitinib, a JAK1/2 inhibitor, or after infection with IAV with an intact NS1 protein. As expected, these conditions were also associated with decreased expression of IFN-signaling genes such as *IFNB1*, *IFNL1*, *STAT1*, and *STAT2*. The JAK proteins are phosphorylated upon binding of type I IFNs to IFN-α receptor 1 (IFNAR1) and IFNAR2, which then activate transcription factors such as STAT1 and STAT2 to induce transcription of ISGs that respond to viral infections (60). The influenza NS1 protein is a known antagonist of the host IFN response and has adapted multiple mechanisms to attenuate type I IFN production by disrupting cellular signaling (57). Other interferon-stimulated lncRNAs have been found to be affected by attenuation of the host immune response. For example, expression of the interferon-stimulated lncRNA *BISPR* was shown to be reduced after ruxolitinib treatment in human epithelial cells as well as in cells with *STAT1* knockdown or inhibition of IRF1, suggesting that *BISPR* can respond to multiple transcription factors during the IFN response (76). Additionally, *BISPR* had increased expression in Huh7 cells infected with influenza virus lacking NS1 compared to the WT virus (76), like the trend observed for *VILMIR*, suggesting that *VILMIR* could be regulated similarly as *BISPR* or other lncRNAs.

Since after suppression of host IFN responses (IAV vs IAVdNS1 infection and pretreatment with ruxolitinib before SARS-CoV-2 infection, Fig. 2) there was a significant decrease in *STAT1* upregulation as well as *VILMIR* upregulation, we predicted that *VILMIR* may be regulated by STAT1. In A549 CRISPRi cells with *STAT1* knockdown, expression of *VILMIR* was slightly reduced; however, this was only significant in one of the two KD lines. While the STAT1 protein was reduced by 85%–95%, we observed a 44%–53% reduction of p-STAT1 in the nucleus, suggesting that there may still be sufficient p-STAT1 available to upregulate ISGs such as *VILMIR*. Therefore, it appears that there may be at least partial regulation of *VILMIR* by STAT1. A *STAT1* KO could help confirm whether *VILMIR* is fully regulated by STAT1, as it is also possible that *VILMIR* is not entirely dependent upon STAT1. The decrease in *VILMIR* upregulation after IAV with an intact NS1 protein as well as after pretreatment with ruxolitinib before SARS-CoV-2 infection (Fig. 2) may be STAT1-independent, as NS1 is known to inhibit the IFN response through many different mechanisms (77), and ruxolitinib specifically binds JAK1 and JAK2 (78). The JAK proteins can also phosphorylate other STAT complexes, such as STAT3 homodimers or STAT2:STAT3 heterodimers, which also have binding motifs within *VILMIR*. Additionally, while JAK/STAT represents the canonical IFN pathway, other non-canonical pathways exist such as MAP kinase and the phosphoinositide 3-kinases/mammalian target of rapamycin pathways that are activated by JAK1/TYK2 and have effects on ISG transcription (60). Furthermore, the NF-κB and IRF pathways can induce ISGs independently of JAK signaling, such as RELB (subunit of NF-κB) and IRF1 which also had predicted binding motifs within *VILMIR*, and several lncRNAs have been found to be regulated by these pathways as well (79, 80). While future studies are needed to elucidate the exact pathway and mechanisms that regulate *VILMIR* expression, *VILMIR* expression was abolished after IFNAR1 KO in Huh7 cells, supporting our hypothesis that *VILMIR* is a novel ISG.

Airway epithelial cells are the first line of defense against respiratory viruses and play an important role in recruiting immune cells to fight infection (81), which is why our initial analysis of *VILMIR* started in epithelial cells. As we have demonstrated, however, *VILMIR* upregulation in response to SARS-CoV-2-infected clinical samples was observed in five immune cell types including macrophages, T cells, mDCs, natural killer cells, and B cells, as well as epithelial cells. In addition to this, it was upregulated after IFN-β treatment in T cell and monocyte cell lines. While many lncRNAs are cell type-specific, the upregulation of *VILMIR* in multiple immune cell types suggests it may play a broader role in immune responses. This also indicates that immune cells need to be investigated to determine the functional impact of *VILMIR in vivo*.

Through CRISPRi knockdown of *VILMIR* expression and RNA-seq-based transcriptome profiling in A549 cells, we found that *VILMIR* KD broadly dampened the host transcriptional response to both IFN-β treatment and IAV H1N1 infection, i.e., genes that were upregulated or downregulated by treatment/infection were still upregulated or downregulated, but with smaller fold changes. Interestingly, during IFN-β treatment, DEGs that were impacted by *VILMIR* knockdown were enriched in Interferon Signaling and JAK/STAT Signaling pathways, and several known ISGs such as *IFIT2* and *IFI44L* were also identified. During H1N1 infection, we also identified known ISGs and immune regulators such as *IFI6* and *CD274*, respectively, that were impacted by *VILMIR* KD. Among the DEGs impacted by *VILMIR* KD, there were several enriched pathways that were shared between IFN-β treatment and H1N1 infection, such as Class I MHC-mediated antigen processing and presentation and ISG15 antiviral mechanism, suggesting that *VILMIR* plays a common regulatory role across the interferon and antiviral response. As viral infection antagonizes many host response pathways apart from interferon, however, we also identified pathways impacted by *VILMIR* KD that were unique to H1N1 infection, such as phospholipid metabolism and cell cycle regulation. Future work is necessary to narrow down this list to genes and pathways directly regulated by or interacting with *VILMIR*.

While the overall magnitude of differential expression changes induced by *VILMIR* KD was relatively small, it may not necessarily reflect less significant biological effect. For example, while lncRNAs generally have low expression levels, several have been found to have significant outcomes on viral replication (82). In addition, the redundancy of ISGs presents a challenge in identifying antiviral functions specific to individual genes, as substantial antiviral response can still be induced in the absence of a specific ISG or even IFN signaling itself (83–85). Another possibility for small fold change differences at the transcription level is that *VILMIR* may function mainly by regulating at the post-transcriptional level, since *VILMIR* was distributed in both the nucleus and the cytoplasm. For example, lncRNA lnc-ISG20 can competitively bind microRNA 326, which in turn decreases the amount of microRNA 326 bound to ISG20 mRNA and therefore enhances the translation of ISG20 (15). LncRNA PYCARD-AS1, also distributed in both nucleus and cytoplasm, can interact with PYCARD mRNA in the cytoplasm and therefore inhibit ribosome assembly for PYCARD translation (86). Finally, while the knockdown of *VILMIR* expression was very efficient, it is important to note that *VILMIR* transcription was not completely abolished, meaning that a low level of the transcript could still have functioned. Alternative approaches such as gene knockout or overexpression may be required for a full *VILMIR* functional perturbation.

Despite VILMIRg2 displaying a slightly higher KD efficiency than VILMIRg1 (Fig. 6), VILMIRg1 KD displayed a higher number of DEGs than VILMIRg2 KD in both the 10 ng/mL IFN-β treatment and H1N1 infection, which most likely also resulted in higher significance values for VILMIRg1 in the enriched pathway analysis. It is possible that this difference in the number of DEGs between VILMIRg1 and VILMIRg2 is due to off-target effects of guide RNAs and/or genetic variation established during the insertion of the guide RNA construct into the genome and subsequent selection. Variation in guide RNA targeting and potential off-target effects is a known challenge of CRISPR systems (87), which is why we exclusively focused on the commonalities between the two KD lines that could be attributed to *VILMIR* KD, including the overall trends and overlapping pathways. Therefore, while some of the changes in gene expression may be attributed to differences between the two KD lines, the observed trend in both KD lines after IFN-β treatment and H1N1 infection is robust, with *VILMIR* KD reducing the overall host transcriptional response to treatment. We also used relatively relaxed criteria for differential expression analysis to observe broader transcriptional impacts of *VILMIR* KD, while future studies would be needed to narrow down the specific genes and pathways directly regulated by *VILMIR*.

In this study, we have identified a previously uncharacterized lncRNA, *VILMIR*, that shows a strong correlation to the host immune response to respiratory viral infection and whose expression impacts the interferon and antiviral response of host genes. We present this lncRNA as a novel ISG that should be further investigated in functional studies. Understanding the regulation of this gene in response to infection and how it functions within the host response may provide new insights into host-pathogen interactions.

## Data Availability

The transcriptomic data discussed in this publication have been deposited in the NCBI Gene Expression Omnibus (GEO) under accession numbers GSE261920 and GSE288251.

## SUPPLEMENTAL MATERIAL

Supplemental figures jvi.00141-25-s0001.docx Figures S1 to S5.

Table S1 jvi.00141-25-s0002.xlsx VILMIR single-cell analysis as seen in Fig. 5A and B.

Table S2 jvi.00141-25-s0003.xlsx RNA-seq analysis of VILMIR knockdown after IFN-β as seen in Fig. 7A.

Tables S3 to S6 jvi.00141-25-s0004.xlsx Pathway and function analysis of VILMIR knockdown after IFN-β shown in Fig. 7.

Table S7 jvi.00141-25-s0005.xlsx RNA-seq analysis of VILMIR knockdown after H1N1 as seen in Fig. 8A.

Tables S8 to S14 jvi.00141-25-s0006.xlsx Pathway and function analysis of VILMIR knockdown after H1N1 shown in Fig. 8.

ASM does not own the copyrights to Supplemental Material that may be linked to, or accessed through, an article. The authors have granted ASM a non-exclusive, world-wide license to publish the Supplemental Material files. Please contact the corresponding author directly for reuse.
