# Supplementary material for: Human long noncoding RNA VILMIR is induced by major respiratory viral infections and modulates the host interferon response
Source: J Virol. 2025 Mar 25;99(4):e00141-25. doi: 10.1128/jvi.00141-25 (PMC11998520; doi:10.1128/jvi.00141-25)
Supplement: Supplemental figures — Figures S1 to S5. [file jvi.00141-25-s0001.docx]

**Supplementary Figures**

**
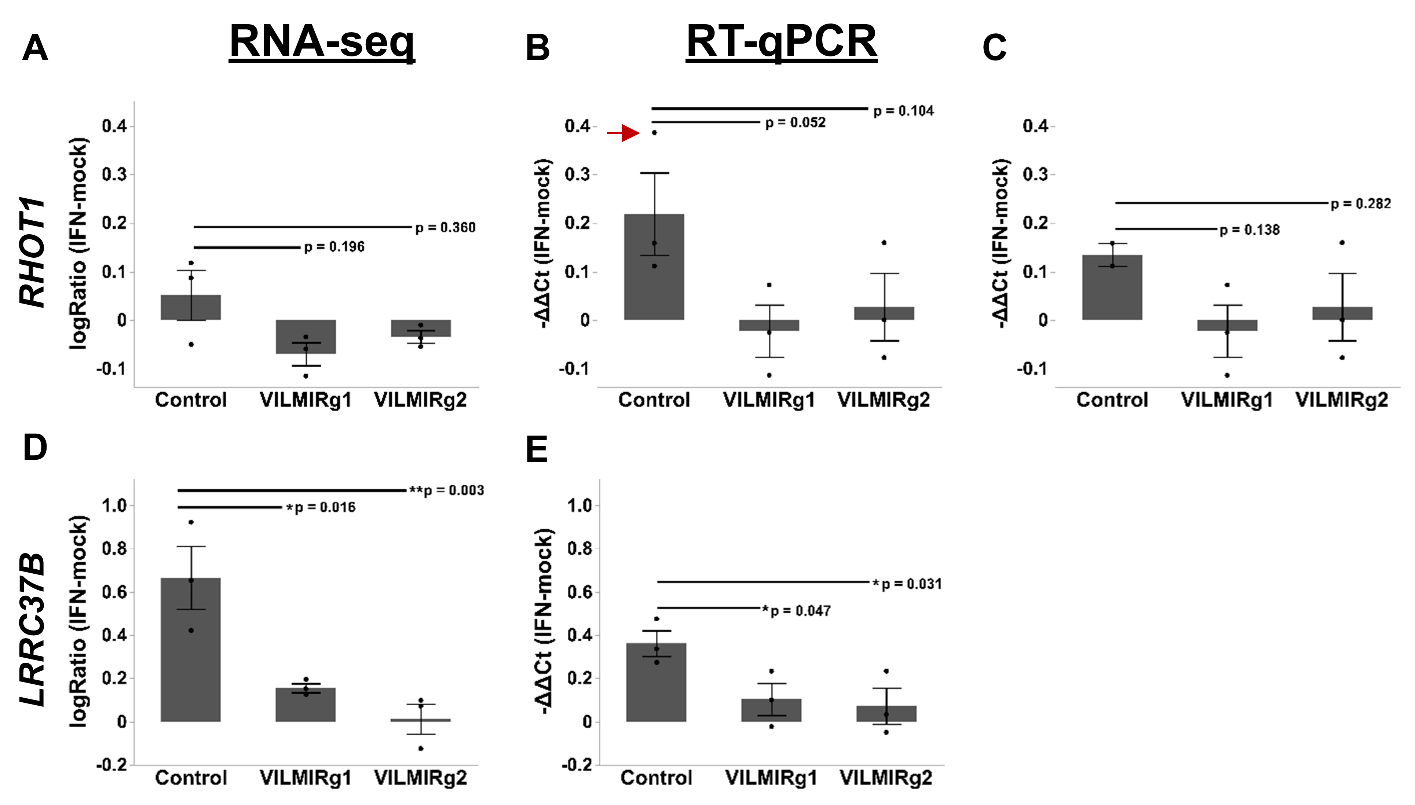
**

**Figure S1. Effect of *VILMIR* knockdown on two immediate neighboring protein-coding genes after** **IFN-β treatment in A549 cells.** A) The expression changes in IFN-β vs. mock treatment ('logRatio (IFN-mock)’) in each A549 cell line from the 10 ng/mL IFN-β treatment determined by the RNA-seq analysis as shown in Figure 7A are shown for *RHOT1* with individual replicates. *P < 0.05, **P <0.01 (unadjusted p-value). B) Relative expression of *RHOT1* was also determined by RT-qPCR and normalized to the mean of the mock-treated samples in each cell line. The data was normalized to GAPDH using the ΔΔCt method. *P < 0.05, **P <0.01 (Student’s t-test). The red arrow indicates one replicate with larger expression changes. C) The same plot as in (B) except removing the potential outlier data point indicated by the arrow in panel B to further demonstrate lack of significance. D-E) Similarly as in A-B, expression changes of *LRRC37B* are shown in both the RNA-seq and RT-qPCR analysis. All plots are expressed as means ± SE (n=3), except for panel C.

**
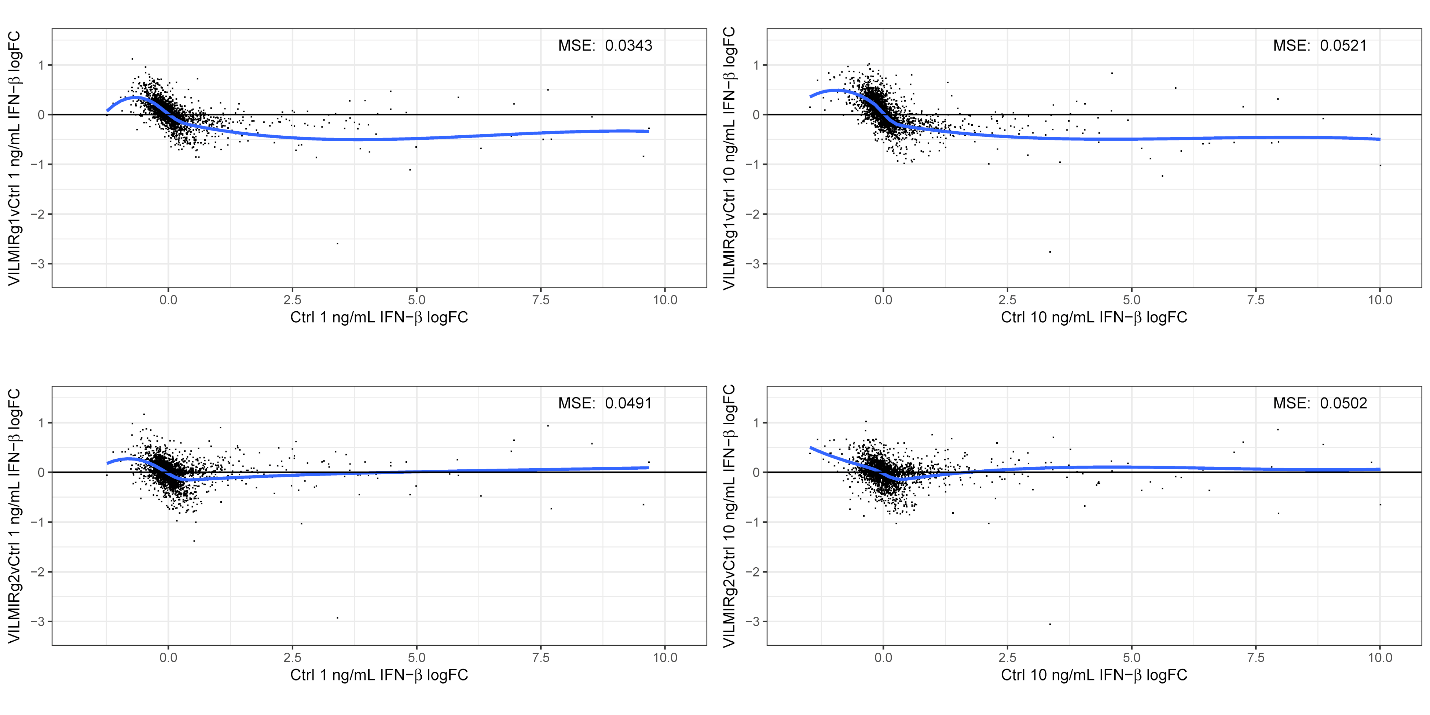
**

**Figure S2. Full plots of the local regression analysis of the impact of *VILMIR* knockdown on IFN-β treatment in A549 cells as seen in Figure 7B.** Scatterplots of the differences between log2FC values in each A549 *VILMIR* KD cell line (VILMIRg1 and VILMIRg2) to the log2FC values in the control cell line (Ctrl) vs. the log2FC values in the control cell line after one of two doses of IFN-β for the same 2,325 differentially expressed genes shown in Figure 7A. The blue line represents the locally estimated scatterplot smoothing (LOESS) curve and the black line at 0 on the y-axis represents if no change was observed after KD (MSE = mean squared error).

**
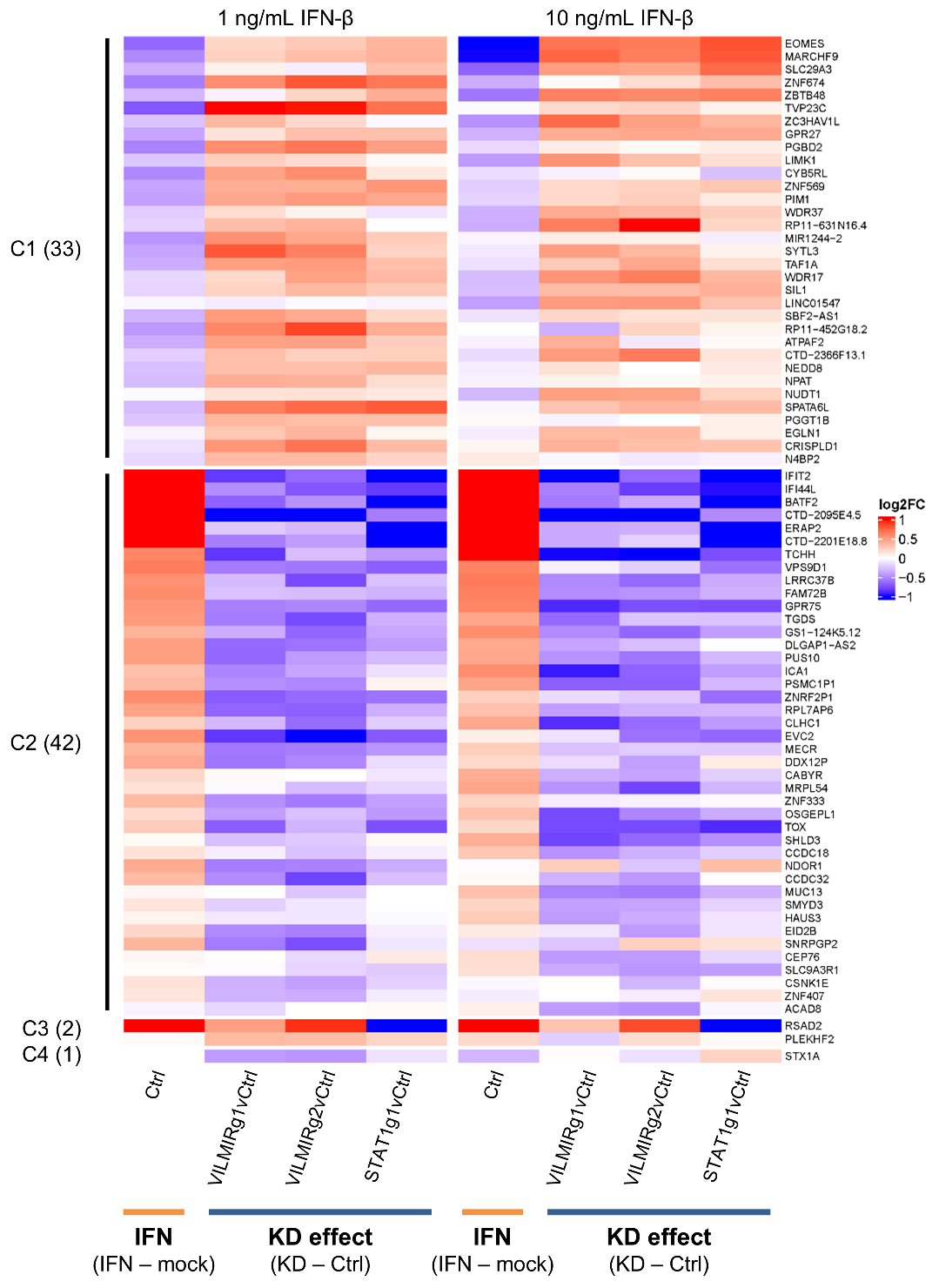
**

**Figure S3. Overview of the subset of 78 genes selected from Figure 7A using the additional filtering criteria described in the main text.** The heatmap is organized the same way as in Figure 7A, but with the corresponding gene names labeled on the right side. See the main text, Figure 7A, Table S2 for additional details.


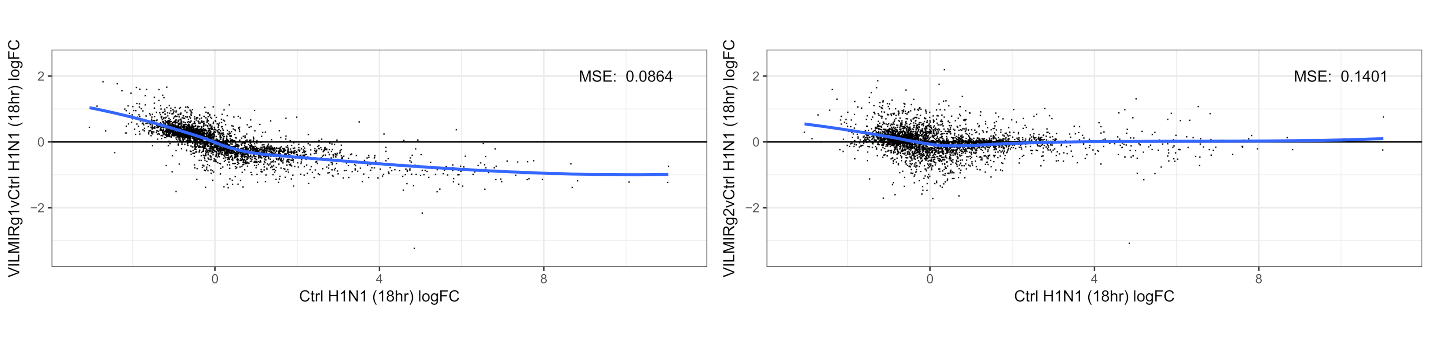


**Figure S4. Full plots of the local regression analysis of the impact of *VILMIR* knockdown on IAV H1N1 infection in A549 cells as seen in Figure 8B.** Scatterplots of the differences between log2FC values in each A549 *VILMIR* KD cell line (VILMIRg1 and VILMIRg2) to the log2FC values in the control cell line (Ctrl) vs. the log2FC values in the control cell line after IAV H1N1 infection for the same 3,068 differentially expressed genes shown in Figure 8A. The blue line represents the locally estimated scatterplot smoothing (LOESS) curve and the black line at 0 on the y-axis represents if no change was observed after KD (MSE = mean squared error).


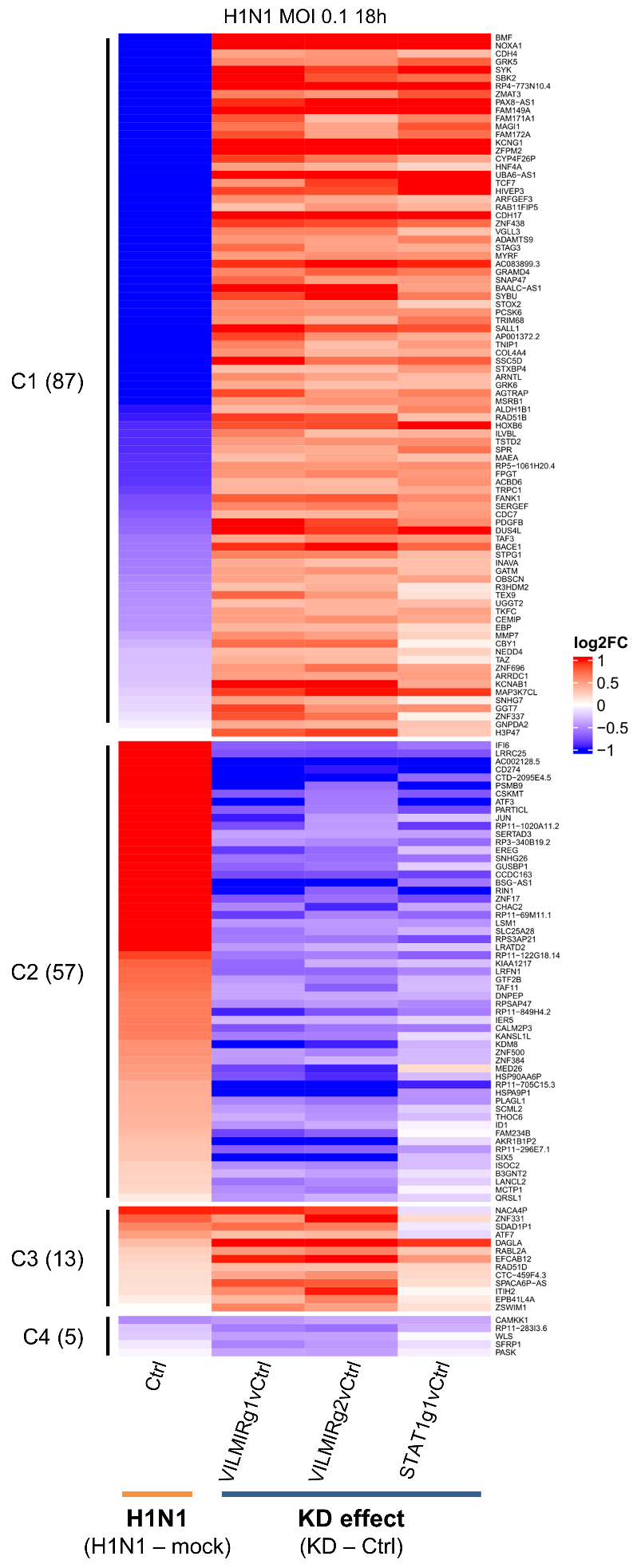


**Figure S5. Overview of the subset of 162 genes selected from Figure 8A using the additional filtering criteria described in the main text.** The heatmap is organized the same way as in Figure 8A, but with the corresponding gene names labeled on the right side. See the main text, Figure 8A, Table S7 for additional details.
